# Supplementary material for: Mesozoic evolution of cicadas and their origins of vocalization and root feeding
Source: Nat Commun. 2024 Jan 8;15:376. doi: 10.1038/s41467-023-44446-x (PMC10774268; doi:10.1038/s41467-023-44446-x)
Supplement: Supplementary file 1 — Supplementary Information [file 41467_2023_44446_MOESM1_ESM.pdf]

Supplementary Information

Mesozoic evolution of cicadas and their origins of vocalization and root feeding

Hui Jiang, Jacek Szwedo, Conrad C. Labandeira, Jun Chen, Maxwell S. Moulds, Bastian Mähler, A. Drew Muscente, De Zhuo, Thet Tin Nyunt, Haichun Zhang, Cong Wei, Jes Rust, Bo Wang

Outline

Supplementary Notes 4

Supplementary Note 1: Description of cicada adult fossils 4

Supplementary Note 1.1: *Eunotalia emeryi* gen. et sp. nov. 4

Supplementary Note 1.2: *Cretotettigarcta problematica* comb. nov. 6

Supplementary Note 1.3: *Cretotettigarcta shcherbakovi* sp. nov. 8

Supplementary Note 1.4: *Vetuprosbole parallelica* (new specimen) 10

Supplementary Note 1.5: *Pranwanna xiai* gen. et sp. nov. 12

Supplementary Note 2: Morphological characters of the Phylogeny 16

Supplementary Note 3: Characters of the Nonmetric multidimensional scaling (NMDS) analysis of cicadoid forewings 21

Supplementary Note 4: Description and measurements of nymphal and exuviae fossils 23

Supplementary Note 4.1: Nymphal species 1– final-instar nymph 23

Supplementary Note 4.2: Nymphal species 2– exuviae 24

Supplementary Note 4.3: Nymphal species 3– exuviae 25

Supplementary Note 4.4: Nymphal species 4– exuviae 26

Supplementary Note 4.5: Nymphal species 5– exuviae 27

Supplementary Note 5: Description for the scheme outlining the forewing venation of Cicadoidea in this study 28

Supplementary Figures 29

Supplementary Figure 1: Details of head, notum, leg, and genitalic structures from adult cicadoid fossils 29

Supplementary Figure 2: Microtomographic reconstruction of *Eunotalia emeryi* gen. et sp. nov. 31

Supplementary Figure 3: Microtomographic reconstruction of *Cretotettigarcta problematica* comb. nov. 33

Supplementary Figure 4: *Cretotettigarcta shcherbakovi* sp. nov. 35

Supplementary Figure 5: New complete female specimen of the *Vetuprosbole parallelica* 36
Supplementary Figure 6: *Pranwanna xiai* gen. et sp. nov. 38
Supplementary Figure 7: Micro-CT reconstruction along the sagittal section of *Pranwanna xiai* gen. et sp. nov., showing internal body structures 40
Supplementary Figure 8: For complete cladograms resulting from the phylogenetic analyses 41
Supplementary Figure 9: Landmarks modeling of the dorsal profile of the Cicadoidea head and notum 43
Supplementary Figure 10: Morphometric measurement data of adults 44
Supplementary Figure 11: Landmark modeling of the Cicadoidea forewing 45
Supplementary Figure 12: Final-instar nymph of Cicadoidea in Kachin amber 46
Supplementary Figure 13: Final-instar exuviae of Cicadoidea in Kachin amber 48
Supplementary Figure 14: Comparisons of forelegs of final-instar cicadoid exuviae from Kachin amber and extant species 49
Supplementary Figure 15: Comparisons of midlegs and hindlegs of final-instar cicadoid exuviae from Kachin amber and extant species 51
Supplementary Figure 16: Schematic line drawings showing forelegs in nymphs of Cicadoidea from mid-Cretaceous to the present 52
Supplementary Figure 17: Cicadoidea fossils from the middle Late Jurassic Yanliao Biota at Daohugou, Inner Mongolia, China 54
Supplementary Figure 18: Abdominal structures associated with the tymbal organs of living species 55
Supplementary Figure 19: Description scheme outlining the forewing venation of Cicadoidea in this study. 57
Supplementary Figure 20: Individual reconstructions of the fossils 59

Supplementary Tables 61
Supplementary Table 1: The description of each landmark for the geometric morphometrics analysis (GMA) of the dorsal profile of the head and thorax 61
Supplementary Table 2: Eigenvalues and contributions of the first five principal components of landmarks for the principal component analysis (PCA) of the dorsal profile of the head and thorax 62
Supplementary Table 3: Eigenvector matrix of the principal components 1, 2 and 3 of the dorsal profile of the head and thorax 63
Supplementary Table 4: Descriptions of each landmark for the geometric morphometrics (GMA) analysis of forewing profiles 64
Supplementary Table 5: Eigenvalues and contributions of the first seven principal components of landmarks of forewing profiles 65

Supplementary Table 6: Eigenvector matrix of the principal components 1and 2 of the forewing profiles 66
Supplementary Table 7: Eigenvalues and contributions of the first three principal coordinates of the nymphal forelegs 67
Supplementary Table 8: Characters of the nymphal forelegs for principal coordinate analysis (PCoA) 68
References 69

Supplementary Notes

Supplementary Note 1. Description of cicada adult fossils

Supplementary Note 1.1 ***Eunotalia emeryi*** gen. et sp. nov. (Supplementary Figure 2)

Description: Length of male body 10.06 mm, width 5.05mm. **Head.** Head width, including eyes, broader than the half the width of the pronotum anterior area (approximate pronotum except the posterior area of pronotal collar or the area before the ambient fissure). Compound eyes large, bulging, and hemispherical in dorsal view; distance between compound eyes slightly less than the diameter of each compound eyes. Vertex with three well-developed ocelli, rounded, and slightly inflated; anteromedian ocelli separated from lateral ocellus by carinate triangular margins; ocellus diameter close to 1/4 diameter of a compound eye. Postclypeus in ventral view bulging, long ellipsoidal, with a distinct median carina and transverse muscle insertion grooves, lateral transverse grooves subparallel; anteclypeus broad in upper aspect and comparatively narrow at lower aspect. Supra-antennal plates visible from ventral view, incised between dorsal surface of postclypeus and margin of compound eye; antennae preserved, with an oblong scape, length/width ratio ca. 2:1; pedicel ovate-oblong, ca. 1/2 length of scape. Rostrum not preserved.

Thorax. Thorax relatively elevated; arched in dorsal and lateral view. Pronotum subhexagonal; overall hue fuscous to dark; pronotum with convex anterior area, forming an open, neck-like feature; paramedian fissures present but not distinct, forming an inverted subtriangular central eminence; lateral fissures absent; ambient fissure developed but mostly connected with paramedian fissures in the central thorax; pronotum concealed all of the mesonotum except scutellum; lacking a wrinkled pattern (Supplementary Figure1a); lateral side of pronotum with one distinct pointed angle, positioned at ca. half the pronotum length; he middle portion of the posterior margin of pronotum almost straight in orientation. Mesoscutellum subtriangular, slightly inflated, extending to approximately tergite II of the abdomen (Supplementary Figure 1a).

Legs. Legs covered with dense setae. Coxae robust. Trochanter scaphoid. Profemur not as broad as among extant cicadids; slightly more robust than meso- and meta femora. Tibia slender compared with femur, tibia apex preserved with small spines of unequal length. Pretarsal claws with lateral setae and fan-like aroliumlia.

Tegmen. Membranous and piliferous in general form. Wing length ca. 10.26 mm; width about 5.19 mm. Costae relatively long and narrow. Nodal incision at ca. 0.64 of wing length. RP separated from vein Sc+R at ca. 0.67 wing length. Sc separated from vein Sc+RA at ca. 0.64 wing length, terminating at nodal incision. RA divided into veins RA_1 and RA_{2+3} at ca. 0.68 wing length, RA_2 and RA_3 separated at ca. 0.77 wing length; and branches into R_4 at ca. 0.82 wing length; RP and M_1 connected by crossvein *r-m* at ca. 0.70 wing length. M branches into veins MA and MP at ca. 0.5 wing length; MA branches into veins MA_{1+2} and MA_{3+4} at ca. 0.66 wing length; MA_{1+2} divided into MA_1 and MA_2 at ca. 0.76 wing length; MA_{3+4} divided into MA_3 and MA_4 at ca. 0.74 wing length; MA_4 and MP_1 connected by crossvein *m* at ca. 0.80 wing length; MP divided into MP_1 and MP_2 at ca. 0.70 wing length. The base of stem CuA strongly curved anteriorly, fused with M for a long distance; stem M+CuA separated from stem R+M+CuA at ca. 0.22 wing length; CuA separated from stem M+CuA at ca. 0. 36 wing length; CuA branches into CuA1 and CuA2 at ca. 0.60 wing length; MP connects with CuA_1 by crossvein *m-cu* at ca. 0.70 wing length; CuA_2 ca. half the length of CuA_1, following the nodal line but slightly separated; CuP and 1A not a clearly fused part or connected by a transverse vein. 1A almost rectilinear and directed rearward and terminates around the nodal clave; 2A curved anteriorly and terminates at inner margin of ca. 0.30 wing length. Nodal line traceable as cross-veins linking RA+Sc, RP, MA, MP, and CuA.

Abdomen. Abdomen subconical, with preserved tymbals. Tymbal subelliptical, slightly convex in the centre, portion of tymbal crinkled. Abdomen filled with mineral matter in burial stage. Sternite VIII sub-rectangular, posterior margin emarginate at the centre.

Genitalia (male). Pygofer dorsal beak absent. Anus developed; anal styles wider than the basal width of anal tube in lateral view. Uncus short. Pygofer style (harpago or paramere) developed, basal style triangular and broad, shrinking upwards and becoming narrow. Other structures not identified.

Supplementary Note 1.2 ***Cretotettigarcta problematica*** (Jiang et al., 2019) comb. nov. (new specimen, Supplementary Figure 3)

2019 *Hpanraais problematicus* Jiang et al: 15, Fig. 1a–l.

2020 *Hpanraais problematicus*: Demers-Potvin et al.: 86, 93, Fig. 5a

Description (for new specimen): Body length 25.29 mm, body width 11.38 mm.

Head. Width of head 2.38 mm, including eyes broader than the half the width of the anterior area of the pronotum (except pronotal collar or the area before ambient fissure). Compound eyes large, bulging, and hemispherical in dorsal view, obovate in lateral view; portion of the head area exposed behind the eyes; distance between compound eyes close to diameter of each compound eye. Vertex with three ocelli, rounded, slightly inflated, anteromedian ocelli separated from lateral ocellus by carinate triangular margins; ocelli diameter distinct, less than the 1/4 diameter of compound eye. Postclypeus in ventral view bulging; width gradually narrows from the upper to lower aspect of the sclerite, with a distinct median carina and transverse grooves of muscles, lateral transverse grooves subparallel; anteclypeus oblong. Antennae intact in male, consisting of 9 segments; supra-antennal plates visible from the ventral view, incised between postclypeus margin and compound eye margin; scape oblong, length/width ca. 2:1; pedicel ovate-oblong, length/width ca. 2:1; first flagellum thinner than pedicel, wider than last flagella; flagellar length gradually reduced from last five to the last flagellum. Rostrum quite long, beyond half of the length of the abdomen.

Thorax. Pronotum subhexagonal; convex anterior area forms an open-neck-like feature; ambient and paramedian fissures present, with a distinct eminence in the central area of the pronotum frontal area; lateral fissures and lateral eminence poorly

developed; pronotal collar distinct with transverse striations and abundant setae, lateral collar area well-developed, the length almost half the length of the pronotum, posterior margin concave in the centre, approaching a W shape along the posterior margin; anterior lateral angle of pronotal collar acuminate, occurring behind half of the length of the pronotum; margin of the pronotal collar from the anterior lateral angle to the middle of the posterior margin forming two obtuse apices, approaching a W shape along each side; margin of pronotal collar from the anterior lateral angle to the head more prominent in the centre than the side. A small portion of the mesonotum exposed and a greater portion of mesonotum concealed by pronotum; the length of the exposed mesonotum shorter than the half of the length of the pronotal collar. Mesoscutellum inflated, the shape approximating an isosceles triangle in dorsal view, rather long, extending to the end of abdomen (Supplementary Figure 1b); the surface ornamented with a regular arrangement of shallow pits. Scutal depression (scutoscutellar sulcus) and base of lateral ridge prominent.

Legs. Legs covered with dense setae. Coxae enlarged; shape resembling a truncated cone; shorter than the femora. Trochanter scaphoid. Profemur elongate and robust; the lower femoral base with a small tooth-like spine, the femoral apex bearing a robust tooth-like spine (Supplementary Figure 1j). Upper medial side of protibia with a blunt spine, the tibial apex displaying a ring of spines with unequal lengths. Metatibia observed with three lateral spines (Supplementary Figure 1l). Meso- and metatarsi preserved with two pairs lateral spines (Supplementary Figure 1m); the second pair of spines more developed than the first pair. Tarsal claw developed with a fan-like arolium, the middle of arolium bearing two petaloid shaped areas of ornamentation.

Tegmen (male). Membranous and piliferous. Wing length as preserved 16.88 mm; width 5.78 mm. Costal relatively long and narrow. Nodal incision at ca. 0.62 wing length. Stem Sc+R+M+CuA slightly curved, Sc+R forked with vein M+CuA at base of the wing length. RP separated from vein Sc+R at ca. 0.35 wing length. Sc separated from vein Sc+RA at ca. 0.58 wing length, terminating at nodal incision. RA forked into three terminals. RA_1 and RA_{2+3} separated at ca. 0.60 wing length, RA_2 and

RA_3 separated from at ca. 0.62 wing length; RP connected with M_1 by crossvein *r-m* at ca. 0.76 wing length. M divided into veins MA and MP at ca. 0.32 wing length; MA divided into veins MA_1 and MA_2 at ca. 0.72 wing length; MA_2 connected with MP by crossvein *m* at ca. 0.74 wing length; MP divided into veins MP_1 and MP_2 at ca. 0.68 wing length. Basal part of stem CuA strongly curved anteriorly; no connecting vein between stem M and stem CuA; CuA divided into CuA_1 and CuA_2 at ca. 0.48 wing length; MP connected with CuA_1 by crossvein *m-cu* at ca.0.70 wing length; length of vein CuA_2 more than half the length of CuA_1; CuA_2 very sinuous, first close to and parallel with a nodal line, then bent at an about right angle and parallel to the tornus, final curved and shifted to the margin; CuP and 1A separated well; CuP almost straight in orientation, ending around nodal clave. 2A curved anteriorly. Nodal line traceable as cross-veins linking RA+Sc, RP, MA, MP, and CuA.

Abdomen. Not visible under light microscopy, obscured by wings. Micro-CT data inferring the abdomen relatively intact, conical, but failed to reconstruct the detailed structures of abdomen and genitalia of this specimen.

Supplementary Note 1.3 ***Cretotettigarcta shcherbakovi*** sp. nov. (Supplementary Figure 4)

Description: Body length 13.67 mm, width 5.97 mm. **Head.** Width of head including eyes broader than the width of the anterior area of the pronotum (except pronotal collar or the area before ambient fissure). Compound eyes large and bulging, hemispherical in in dorsal view, obovate in lateral view; portion of the head area exposed behind the eyes; distance between the compound eyes close to the diameter of each compound eye. Vertex with three ocelli, rounded and slightly inflated, anteromedian ocellus separated from lateral ones by carinate triangular margins; ocelli diameter distinct, less than the 1/4 diameter of compound eye. Face not clearly visible. Left antennae intact but only observed last 7 segments. Rostrum very long, beyond the half length of abdomen.

Thorax. Pronotum subhexagonal, convex front area forms an open-neck-like feature; paramedian fissures developed, with a distinct eminence in the central area of pronotum frontal area; lateral fissures and lateral eminence poorly developed; pronotal collar distinct with transverse striations and abundant setae, length of pronotal collar almost equal to the half length of the pronotum, posterior margin concave not distinct in centre; anterior lateral angle of pronotal collar obtuse, occurring behind half the length of the pronotum; margin of pronotal collar from the anterior lateral angle to the middle of the posterior margin forming two obtuse apices, approaching a W shape along each side; lateral collar developed, the width of anterior lateral collar broader the width of neck-like edge of anterior area of pronotum; lateral margin of pronotal collar from the anterior lateral angle to the head arched. Mesonotum exposed small portion except scutellum and much of mesonotum concealed by pronotum; length of the exposed mesonotum (except mesoscutellum) shorter than the half length of the pronotal collar. Mesoscutellum inflated, the shape close to an isosceles triangle in dorsal view, extremely long, extending to the end of abdomen (Supplementary Figure 1c); the surface ornamented with a regular arrangement of shallow pits; scutal depression (scutoscutellar sulcus) and base lateral ridge prominent.

Legs. Legs covered with dense setae; coxae enlarged, shape like a truncated cone, shorter than the femur. Trochanter scaphoid. Profemur elongate and robust, apex of femur with a robust tooth-like spine (Supplementary Figure 1k). Upper medial side of protibia with a blunt spine, the apex of tibia with a ring of unequal length spines. Protarsus without lateral spine (Supplementary Figure 1n); mesotarsi and metatarsi preserved two pairs lateral spines (Supplementary Figure 1o); the second pair of spines more developed than the first pair. Claw developed with fan-like arolium, and the arolium with a two-petal ornamentation.

Tegmen (male). Membranous and piliferous. Wing length as preserved 11.15 mm; width 3.90 mm. Costa relatively long and narrow. Nodal incision at ca. 0.65 wing length. Stem Sc+R+M+CuA slightly curved, Sc+R separated with stem M+CuA

at ca. 0.27 wing length. RP separated from vein Sc+R at ca.0.39 wing length. Sc separated from vein Sc+RA at ca. 0.61 wing length, ends at nodal incision. RA divided into RA_1 and RA_{2+3} at ca. 0.57 wing length, separated RA_3 at ca. 0.75 wing length; RP and M_1 connected by crossvein *r-m* at ca. 0.77 wing length. M divided into MA and MP at ca. 0.36 wing length; MA divided into veins MA_1 and MA_2 at ca. 0.75 wing length; MA_2 and MP connected by crossvein *m* at ca. 0.76 wing length; MP divided into veins MP_1 and MP_2 at ca. 0.69 wing length. The basal portion of stem CuA strongly curved anteriorly. No connected vein between stem M and stem CuA; CuA divided into CuA_1 and CuA_2 at ca. 0.59 wing length; MP and CuA_1 connected by crossvein *m-cu* at 0.68 wing length; CuA_2 short and straight, terminating at nodal clave; CuP and A_1 fused a short distance, and then almost oriented straight, into nodal clave. 2A curved anteriorly and terminated at the inner margin. Nodal line traceable as cross-veins linking RA+Sc, RP, MA, MP, and CuA.

Abdomen. Abdomen relatively intact, subconical, with preserved tymbal. Tymbal suborbicular, slightly convex in the middle, portion of tymbal crinkled.

Genitalia. Male. The details reconstructed by micro-CT vague, and unrecognizable for the time being.

Supplementary Note 1.4 ***Vetuprosbole parallelica*** Fu, Cai and Huang, 2019 (new specimen, Supplementary Figure 5)

Description (for new specimen): Body length 17.58 mm, width 6.67 mm. **Head.** Not preserved. **Thorax.** Pronotum subhexagona, convex front area forms an open-neck-like feature; paramedian fissures developed, with a distinct eminence in the anterior central area of pronotum; lateral fissures and lateral eminence poorly developed; pronotal collar distinct with transverse striations, the length shorter than the half length of the pronotum, posterior margin almost straight in the middle part; lateral collar developed; the width of anterior lateral collar close to the width of neck-like edge of pronotum; anterior lateral angle of pronotal collar obtuse, occurring behind half the length of the pronotum. Mesonotum considerably exposed (except

mesoscutellum) and small portion of mesonotum concealed by pronotum, the length of the exposed mesonotum slightly shorter than the length of pronotum. Mesoscutellum inflated, the shape close to an isosceles triangle in dorsal view, extending to around tergites II of abdomen, the surface with wrinkles and folds (Supplementary Figure 1d), Scutal depressions (scutoscutellar sulcus) and base lateral ridge prominent.

Legs. Legs covered with dense setae; coxae robust, truncated cone shape-like, shorter than the femur. Trochanter scaphoid. Femur elongate and robust. The apex of metatibia with unequal length spines. Meso and metatarsi preserved two pairs lateral spines. The second pair of spines more developed than first pair. Claw developed with fan-like arolium (Supplementary Figure 1p). Meracanthus reaching to tergites II of abdomen (Supplementary Figure 5b).

Tegmen (female). Membranous and piliferous. Wing length as preserved 18.92 mm; width 7.70 mm. Costa relatively long and narrow. Nodal incision at ca. 0.61 wing length. RP separated from vein Sc+R at ca. 0.24 wing length. Sc separated from vein Sc+RA at ca. 0.56 wing length, terminating at nodal incision. RA divided into veins RA_1 and RA_2 at ca. 0.56 wing length; RP and M_1 connected by crossvein *r-m* at ca. 0.66 wing length. M separated from M+CuA at ca. 0.41 wing length, and then divided into veins MA and MP at 0.29 wing length; MA divided into veins MA_1 and MA_2 at ca. 0.63 wing length; MA_2 and MP connected by crossvein *m* at 0.67 wing length; MP divided into veins MP_1 and MP_4 at ca. 0.53 wing length. The basal of stem CuA strongly curved anteriorly; CuA divided into CuA_1 and CuA_2 at 0.46 wing length; MP and CuA_1 connected by crossvein *m-cu* at ca. 0.59 wing length; CuA_2 very sinuous, parallel with a nodal line, bent at about right angle and parallel to the tornus, then curved and shifted to the margin; CuP and 1A fused a short distance at base, and then almost oriented straight, end at nodal clave. 2A curved anteriorly. Nodal line traceable as cross-veins linking RA+Sc, RP, MA, MP, and CuA.

Hindwing. Membranous, incomplete. RA and RP single; RP and RA connected by crossvein *rp-ra*. Vein M divided into MA and MP; MA single and MP divided into

but in left hindwing divided into four terminals; in left hingwing, MA divided into two terminals. RP and MA_1 connected by crossvein *rp-m$_1$*; MP and CuA_1 connected by crossvein *m$_3$-cua$_1$*. CuA divided into CuA_1 and CuA_2; he separation of CuA earlier than the separation of M, the separation of M around the half length of CuA_1; Part of CuP and 1A preserved and observed from the left hindwing.

Abdomen. Abdomen subcylindrical. Tymbal not preserved. Sternite VII approximate inverted trapezoid, lateral margin curved forming two obtuse apices, posterior margin with the emarginate (Fig. 3m).

Genitalia (female). Gonocoxite XIX, part of ovipositor and ovipositor sheath preserved and observed in ventral view.

Supplementary Note 1.5 *Pranwanna xiai* gen. et sp. nov. (Supplementary Figure 6)

Description: Male body of holotype species length about 5.45 mm, width 2.21 mm. The female body of holotype species length 7.03 mm, width 2.73 mm. The male body of paratype species length about 5.45 mm, width 2.22 mm. **Head.** Head covered with hairs, the width including eyes broader than the width of the anterior area of the pronotum (except pronotal collar or the area before ambient fissure). Compound eyes large and bulking, hemispherical in dorsal view, obovate in ventral view; part of the head area is exposed behind the eyes. Vertex with three well-developed ocelli, rounded and slightly inflated, anteromedian ocellus separated from lateral ones by carinate triangular margin; the diameter ratio of compound eye and ocellus close to 4:1. Postclypeus in ventral view bulging, the width gradually narrow from the top-down, with distinct median carination and transverse grooves of muscles, lateral transverse grooves subparallel (Supplementary Figure 1h); anteclypeus elliptical; the antennae intact in male with 9 segments, and its length close to the length of the head; six segments observed in female; supra-antennal ledges visible from the ventral view, incised between dorsal surface of postclypeus and margin of compound eye; scape oblong, length/width about 2:1, broader than pedicel; pedicel ovate-oblong, length/width about 2:1; flagella slender, the length similar from first flagellum to the

fourth flagellum, the length gradually shorter from the five flagella to the end flagellum. Rostrum (with black apex in male) reaching metacoxa legs.

Thorax (Supplementary Figure 1e, f)**.** Pronotum sub-trapezoidal, covered with small setae; pronotum convex front edge forms an open-neck-like feature; paramedian, lateral and ambient fissures developed, forming distinct eminences in the central and lateral area of anterior area of pronotum; pronotal collar with transverse striations and abundant hairs, gradually widening from lateral to middle; the middle of posterior margin concave, resembling W-shape of the entire posterior margin; the anterior angle outwards on both sides, the anterior angle of pronotum collar of male more rounded and blunt than those of female; the length of the collar almost half the length of the pronotum; fuscous coloration pattern on the anterior part of pronotum, lateral eminences and pronotal collar in male; in female, fuscous pattern observed in the area of pronotum before ambient fissures except the front "neck" area and eminences. Mesonotum exposed small portion except scutellum, and much of mesonotum concealed by pronotum, the length of the exposed mesonotum slightly shorter than the half the length of the pronotum, close to the 1/2 length of pronotum. Exposed submedian sigilla white to silver in male of holotype specimen. Mesoscutellum distinctly inflated, slightly arched in profile view, the shape close to an isosceles triangle in dorsal view, the distal margin relatively round, the surface ornamented with regular arrangement of shallow pits and fine setae. Scutal depression and base of lateral ridge prominent, lateral ridge gradually moderate and extends in an arc along the mesonotum to submedian sigilla. Two dark brown bands on the surface of the scutellum in male, and the bands widened gradually from the centre to both sides, the band pattern not observed in female; the distal margin of the scutellum in female slightly wider than that in male. Meracanthus quite long in male, beyond half the length of tergites II of abdomen; meracanthus in female reaches approximately tergite II of abdomen. No developed and dilated opercula.

Legs. The legs covered with dense setae. Coxae robust. Trochanter scaphoid. Femur elongate and robust; the profemur stronger than the meso and metafemur. Tibia

slender compared with femur, the apex of tibia preserved short spines. Pretarsal claws with lateral seta and fan-like arolium.

Tegmen (male): membranous and piliferous. Wing length of holotype male specimen as preserved 5.66 mm, width 2.23 mm; wing length of paratype male specimen as preserved 5.41mm, width 2.28 mm. Costa relatively long and narrow. Nodal incision at of about ca. 0.66 wing length. Stem Sc+R+M+CuA slightly curved, Sc+R forked with vein M+CuA at base of the wing length. RP separated from vein Sc+R at ca. 0.50 wing length. Sc separated from vein Sc+RA at ca. 0.54 wing length, terminating at nodal incision. RA divided into veins RA_1 and RA_{2+3} at ca. 0.65 wing length, divided into RA_3 at ca. 0.72 wing length; RP and M_1 connected by crossvein *r-m* at ca. 0.79 wing length. M separated from M+CuA at ca. 0.33 wing length, and then divided into veins MA and MP at about ca. 0.47 wing length; MA divided into veins MA_1 and MA_2 at about 0.66 wing length; MA_2 and MP connected by crossvein *m* at about 0.72 wing length; MP single. CuA divided into CuA_1 and CuA_2 at ca. 0.52 wing length; MP and CuA_1 connected by crossvein *m-cu* at ca. 0.63 wing length; CuA_2 very short, terminates at nodal clave; CuP and 1A fused a short distance at very base, and then almost oriented straight; CuP terminates at nodal clave. 2A curved anteriorly and terminates at inner margin of about ca. 0.31 wing length. Nodal line traceable as cross-veins linking RA+Sc, RP, MA, MP, and CuA. Marginal membrane very narrow.

Tegmen (female): membranous and piliferous. Wing length as preserved 7.24 mm; width 2.63 mm. Costal relatively long and narrow. Nodal incision at about ca. 0.64 wing length. Stem Sc+R+M+CuA slightly curved, Sc+R forked with vein M+CuA at base of the wing length. RP separated from vein Sc+R at ca. 0.50 wing length. Sc separated from vein Sc+RA at ca. 0.56 wing length, terminates at nodal incision. RA divided into veins RA_1 and RA_{2+3} at ca. 0.64 wing length, RA_3 separated from RA_{2+3} at about ca. 0.70 wing length; RP and MA_1 connected by crossvein *r-m* at about ca. 0.70 wing length. M separated from M+CuA at about ca. 0.41 wing length, and then divided into veins MA and MP at ca. 0.50 wing length; MA divided into veins MA_1

and MA_2 at about ca. 0.68 wing length; MA_2 and MP connected by crossvein *m* at about ca. 0.70 wing length; MP single. CuA divided into CuA_1 and CuA_2 at ca. 0.5 wing length; MP and CuA_1 connected by crossvein *m-cu* at ca. 0.66 wing length; CuA_2 very short, terminating at nodal clave; CuP and 1A fused a short distance, and then almost oriented straight, nodal clave. 2A curved anteriorly and terminated at inner margin of about ca. 0.31wing length. Nodal line traceable as cross-veins linking RA+Sc, RP, MA, MP, and CuA.

Hindwing. The hindwings membrane, covered by forewings and partial veins observed. RA and RP single; RP and RA connected by crossvein *rp-ra*. MA divided into MA and MP, MA and MP single. CuA divided into CuA_1 and CuA_2. RP and MA_1 connected by crossvein $rp\text{-}m_1$. MP and CuA_1 connected by crossvein $m_3\text{-}cua_1$. Part of CuP observed from lateral view.

Abdomen. The abdomen relatively intact, subcylindrical, with silvery small pores and pile. The length of abdomen close to that of the thorax. Male and female specimen both preserved a tymbal. Tymbal suborbicular, slightly convex in the middle (Fig. 2t, u); male with robust tymbal muscle and developed abdominal cavity. Internal structure of abdomen such as malpighian tubule preserved in the type species of the male (Supplementary Figure 7); tergites II to VIII in dorsal view and sternite II to VII in ventral view with fuscous band about 2/3 area and narrowed silver to yellow band about 1/3 area in posterior margin. Sternite VII of male sub-rectangular. Sternite VIII approximate inverted trapezoid, lateral margin curved forming two obtuse apices, posterior margin with the emarginate in middle more distinct in male than that in female. The sternite VIII longer in male.

Genitalia (male). Pygofer dorsal beak absent. Anus developed, anal styles wider than the basal width of anal style in lateral view, surface with circle patterns. Uncus not observed (very short or absent). Upper lobe moderately developed, rounded. Basal lobe developed, projecting outwards, basically triangular but the distal aspect rounded in lateral view. In lateral view, pygofer style (harpago or paramere) developed, basal triangular and broad, shrinking upwards and becoming narrow, distal aspect expanded

slightly. Aedeagus fuscous, tubular with recurved endotheca supporting membrane, distal part broadened in lateral view. **Genitalia (female)**. Covered in dense silvery pile. Dorsal beak absent. Anal styles developed, wider than the width of ovipositor sheath. Gonocoxite XIX visible in lateral view. Distal part of ovipositor fuscous, still wrapped in the ovipositor sheath, distal part.

Supplementary Note 2. Morphological characters of the phylogeny

1. Number of ocelli: (0) three; (1) two
2. Distance between compound eyes: (0) shorter than the diameter of compound eye; (1) longer than the diameter of compound eye
3. Diameter of ocelli: (0) close to 1/4 the diameter of the compound eye; (1) shorter than 1/4 the diameter of the compound eye
4. Antenna segmented: (0) yes; (1) no
5. Length of mouthparts: (0) not reaching to metacoxa; (1) nearly or at least to the metacoxa
6. Part of head can be visible in dorsal view: (0) no; (1) yes
7. Width of the head: (0) more than 1/2 the width of the front half of the pronotum; (1) not more than 1/2 the width of the front half of the pronotum
8. Postclypeus: (0) with longitudinal median carination (1) median relatively flat (2) with longitudinal median sulcus
9. Anterior margin, the part between two compound eyes, of pronotum: (0), relatively smooth; (1) with an angle
10. Pronotum: (0) largely concealing the part of mesonotum (except scutellum or cruciform elevation); (1) not covering all part of mesonotum (except scutellum or cruciform elevation)
11. Paramedian fissure: (0) not distinct; (1) distinct
12. Lateral fissure: (0) absent; (1) present
13. Lateral fissure: (0) not distinct; (1) distinct

14. Pronotal collar (with distinct transverse wrinkle pattern): (0) not distinct; (1) distinct
15. Length of pronotum collar: (0) more than 1/2 the length of the pronotum; (1) less or close to or no more than 1/2 the length of the pronotum
16. Length of pronotum: (0) longer than exposed part of mesonotum (except scutellum or cruciform elevation); (1) shorter than exposed part of mesonotum (except scutellum or cruciform elevation)
17. Lateral collar of the anterior portion of pronotum (around the paramedian fissure): (0) developed; (1) undeveloped
18. Shape of the middle part of posterior margin of pronotum: (0) approximately straight or slightly concave in the middle; (1) semicircular
19. Posterior lateral angle of pronotum: (0) distinctly not protruding backward; (1) distinctly protruding backward
20. Mesonotum: (0) with scutellum; (1) with cruciform elevation
21. Anterior lateral angle of pronotum: (0) located at the middle half of the length of the pronotum; (1) located at the latter half of the length of the pronotum; (2) Located at the front half of the length of the pronotum
22. Length of scutellum: (0) extended to the position around sternites I–III of the abdomen; (1) extend to the position around the end of genitalia
23. Length of scutellum and pronotum: (0) shorter than the length of pronotum; (1) no less than the length of pronotum; (2) similar in length
24. Length of scutellum (cruciform elevation) compared with the length of exposed mesonotum (the area before scutoscutellar sulcus): (0) almost equal to the length of exposed scutellum; (1) longer but not equal to the length of exposed scutellum; (2) shorter than the exposed part of mesonotum (the area before scutoscutellar sulcus)
25. Thoracic ganglia: (0) separated; (1) fused
26. Opercula: (0) absent; (1) present

27. The pronotum covers a small portion of the anal area of the forewing when the wings are retracted to the sides of the body: (0) no; (1) yes
28. Fold of costal area: (0) present; (1) absent
29. Width of costal area: (0) obviously shorter than 1/3 of the wing width; (1) close to 1/3 of the wing width
30. Widest distance between Sc and C veins: (0) wider than the distance between RP and RA; (1) thinner than the distance between RP and RA
31. Separation of vein RP: (0) not near the nodal line; (1) near the nodal line
32. Basal cell: (0) CuA and M connected; (1) CuA and M very close but not connected
33. Number of RP terminals: (0) one; (1) two; (2) more than two
34. Number of RA terminals: (0) 1; (1) 2; (2) 3; (3) 4; (4) more than four
35. Basal cell length: (0) longer or similar to the length of stem RA; (1) stem RA at least longer than the twice the length of the basal cell
36. Separation of vein M: (0) not near the nodal line; (1) near the nodal line
37. Bifurcations of M and RP from R: (0) separated at the anterior and posterior part of the forewing, respectively; (1) both separated at the anterior part of the wing
38. Level of vein MP bifurcation: (0) no; (0) 1; (1) 2; (2) 3
39. Number of MA terminal: (0) 1; (1) 2; (2) 3; (3) 4
40. Number of M terminals: (0) 2; (1) 3; (2) 4; (3) 6 (4) more
41. Vein M (0) separated from R first and then separated from CuA; (1) separated from CuA first and then separated from R; (2) almost separate at the same time.
42. Whether M and CuA fused a distance after the basal cell: (0) no; (1) yes
43. End of CuP: (0) longer beyond half the length of the forewing; (1) nearly as long at half the length of the forewing; (2) not terminated at half the length of the forewing
44. Claval veins 2A (A_2 in previously description of cicadoid fossils) and forewing margin: (0) well separated through most of the length; (1) fused at much of their length (or absent)

45. Forewing vein CuA_1 division (most common state): (0) such that proximal portion shortest; (1) such that proximal portion longest
46. Comparisons of the length of CuA_1 and CuA_2: (0) the length of CuA_2 shorter than 1/2 the length of CuA1; (1) the length of CuA_2 longer than 1/2 the length of CuA_1
47. Vein CuA2 bent to the distal direction: (0) yes; (1) no
48. Separation of vein M: (0) after the separation of vein RP from R; (1) before the separation of vein RP from R
49. Vein CuP and 1A: (0) well separated; (1) very close to each other (or fused at portion more than 1/2 the length of 1A)
50. With two distinct veins between the whole clade of M and RP: (0) yes; (1) no
51. Nodal line: (0) absent; (1) present
52. Nodal line: (0) distinct; (1) indistinct
53. Middle part of nodal line position: (0) at the middle of the wing; (1) at posterior half part of wing; (2) at anterior half part of the wing
54. Angle of anal area of the wing: (0) higher than the cubital angle (tornus) or absent; (1) lower (or similar) than the cubital angle
55. Tegmen: (0) membranous; (1) punctate
56. Long spine at apex of prefemur: (0) no; (1) yes
57. Profemoral primary spine: (0) erect; (1) lying flat, prostrate
58. Profemur thickening (compared with meso and metafemur): (0) indistinct; (1) distinct
59. Protibia with spine at the upper inner part: (0) absent; (1) present
60. Tarsi: (0) with arolium; (1) with empodium; (2) with seta
61. Metatarsus: (0) presents a row of spines; (1) presents two lateral spines of each of the first two segments; (2) absent or distinct spines not observed
62. The tymbal of female: (0) present; (1) absent or indistinct
63. Tymbal muscle: (0) undeveloped; (1) developed
64. Male tymbal cover: (0) without; (1) with
65. Abdominal (resonant?) cavity of male: (0) absent; (1) present

66. Tympana: (0) absent; (1) present
67. Female accessory glands of common oviduct: (0) absent; (1) present
68. Testes: (0) located anteriorly, centered over abdominal sternites II–III; (1) located posteriorly, centered over abdominal sternite VI
69. Sternite VIII shielding genitalia: (0) not shielding; (1) shielding
70. Male subgenital plate: (0) lacking; (1) present
71. Pygofer with basal lobe: (0) absent; (1) present
72. Pygofer upper lobe: (0) absent; (1) present
73. Pygofer distal shoulder: (0) undeveloped; (1) well-developed
74. Genital apertures: (0) monotrysian; (1) ditrysian
75. Genital style: (0) present; (1) absent
76. Basal plate of aedeagus: (0) undivided; (1) reduced
77. Ventrobasal pocket of aedeagus: (0) present; (1) without
78. Uncus: (0) distinct; (1) absent or very small
79. Uncus: (0) not retractable within pygofer; (1) retractable within pygofer
80. Clasper: (0) absent; (1) present
81. Apical part of theca: (0) without leaf-like lateral lobes; (1) with a pair of leaf-like lateral lobes; (2) with sclerotized flanges

Supplementary Note 3. Characters of the Nonmetric multidimensional scaling (NMDS) analysis of cicadoid forewings

1. The number of the terminals of RA: (0) 2; (1) 3; (2) 4 (3) more than 4
2. The number of the terminals of RP: (0) 1; (1) 2; (2) more
3. The number of the terminals of MA: (0) 2; (1) 3; (2) 4; (3) more than 4
4. The number of the terminals of MP: (0) 1; (1) 2; (2) 3; (3) 4; (4) more than 4
5. Whether RA_1 is close to the nodal line: (0) yes; (1) no
6. The order of the forking point of RP separates from R and the forking point of M separates: (0) RP earlier; (1) M earlier
7. Whether the forking point of RP separated from R is close to the forking point of M separated at the wing length: (0) no; (1) yes
8. Whether the separation of RP is close to the nodal line: (0) no; (1) yes
9. Whether the separation of M is close to the nodal line: (1) yes; (0) no
10. The junction of M and CuA: (0) point or very short fusion; (1) long fusion; (2) no connection; (3) small transverse vein
11. Whether CuA is fused a small distance along the nodal line: (0) yes; (1) no
12. Whether CuA_2 is tortuous: (0) yes; (1) no
13. Length comparisons of the lengths of CuA_2 and CuA_1: (0) the length of CuA2 is no less than half the length of the CuA_1; (1) the length of CuA_2 is less than half the length of the CuA_1
14. Whether CuA_2 occurs along the nodal line: (0) no; (1) yes or very close
15. The number of the terminals of CuA_2: (0) 1; (1) 2; (2) more than 2
16. Whether CuP is separated from 1A: (0) yes; (1) no
17. Whether 2A is separated from the margin: (0) yes; (1) no
18. Whether the costal area has a fold: (1) yes; (0) no
19. The position of nodal line is: (0) posterior; (1) middle; (2) anterior of the wing
20. The portion of nodal line between RP and CuA is: (0) relatively flat; (1) going forward
21. Whether the penultimate terminal of RA is close: (0) no; (1) yes

22. Whether the nodal line is distinct: (0) yes; (1) no
23. Whether there is a crossvein between RP and MA besides the nodal line: (0) no; (1) yes
24. Comparison of the lengths of Ms (=stem M) and Ma (=the part of MA before the connection between M2 and the crossvein ma-rp /nodal line): (0) Ms > Ma; (1) Ms =Ma; (2) Ms <Ma
25. Comparison of the lengths of Ms and Rh (crossvein m-r/nodal line connected MA and RP): (0) Ms> Rh; (1) Ms =Rh; (2) Mg <Rh
26. Comparison of the lengths of Ma and Rh: (0) Ma > Rh; (1) Ms =Rh; (2) Ma <Rh
27. Basal part of CuA curved very much: (0) no; (1) yes
28. The position where the transverse vein intersects with CuA_1 makes CuA_1: (0) distal longer of CuA_1; (1) middle of CuA_1; (2) proximal longer of CuA_1
29. The end of CuP: (0) longer beyond half the length of the forewing; (1) nearly as long at half the length of the forewing; (2) not terminated beyond half the length of the forewing
30. Comparison of the lengths of the widest distance between C to Sc and the widest distance between 1A vein and wing margin: (0) the former longer than the later; (1) approach each other; (2) the later longer than the former
31. The ends of CuP and 1A separated: (0) not distinct; (1) distinct
32. The angle of anal area of the wing: (0) higher than the cubital angle (tornus) or absent; (1) lower (or similar) than the cubital angle

Supplementary Note 4. Description of nymphal and exuviae fossils

The descriptive terminology for the structures and the measurement terminology are based on Maccagnan and Martinelli, 2004; Moulds, 2005; Hou et al., 2014; and Song et al., 2019. **Measurement terminology**: Body length (BL), head width (HW), postclypeus length (PCL), postclypeus width (PCW), pro-mesonotum length (PML), pronotum length (PL), pronotum width (PW), wing length (WL), Profemur length (FL), Protibia length (TL), and femoral tooth angle (FA).

Supplementary Note 4.1 Nymphal species 1, final-instar nymph (NIGP201898) (Supplementary Figure 12).

In dorsal view, crown preserved with one intact compound eye (Supplementary Figure 12c). Antenna filiform and segmented; preserved ten segments (Supplementary Figure 12g); most of scape concealed in the antennal fovea; pedicel elongate, about twice times longer than width; eight flagellomeres preserved; the length of the scape and pedicel slightly longer than the five segments of the flagellum, the diameter of the last two segments similar. Postclypeus and anteclypeus prominently inflated (Supplementary Figure 12e); postclypeus preserved incomplete, missing the upper part, postclypeus present, subobovate shape in ventral view; the postclypeus and anteclypeus tightened and narrowed at the location of their connection; the anteclypeus contracts at both ends and expands in the middle. Rostrum reaching to metacoxae (Supplementary Figure 12d, e). The anterior area of the pronotum surrounded by two, prominent, paramedian fissures, and the anterior margin of the pronotum slightly narrower than the width of the head in dorsal view (Supplementary Figure 12f). The left side of the body deformed and contracting downward. The length of the mesonotum close to the length of the pronotum, arc-shaped along the middle of the posterior margin of the mesonotum (Supplementary Figure 12f). Forewing bud developed and conspicuously reaching laterally to the middle of second abdominal segment (Supplementary Figure 12c, d); part of hind wing bud concealed under the forewing bud, a fraction visible (Supplementary Figure 12c, d). Foreleg specialized (Supplementary Figure 12h–l).

Coxa of all legs brawny and elongated; trochanter elongated and elbow-like. Profemur enlarged, powerful and slightly bent forward; with long and sharp posterior tooth; accessory tooth slightly smaller than intermediate tooth, accessory, and intermediate teeth robust and with a low cusp on distal edge; femoral comb preserved with five teeth, the comb's first tooth relatively more independent and slightly larger than the other teeth; the last and the penultimate tooth of femur comb more aggregated. Protibia arched and sickle-shaped; apical tooth long and sharp; the point of tibial blade small, separated from the tibial apical tooth by a small incision, the length of the secondary apical tooth of tibia no more than 1/2 of the length of the apical tibial tooth; the blade of tibia developed. Meso and metafemur slightly bent, preserved without deformation (Supplementary Figure 12c). The central part of metatarsi with first half slightly bent and shrunk; the apex of metatibia with five spines distinctly visible, one of them longer and dominant, presently needle-shaped (Supplementary Figure 12m, n, p). Tarsus of all legs with two segments; pretarsi of all legs swollen, right meta-pretarsi preserved as an unequally sized claw (Supplementary Figure 12m–p). Abdomen elongate, approximately cylindrical in cross-section, ten sternites visible. Genitalia indistinguishable.

Measurement (mm or degree). BL 23.1, HW 4.1, PCL 4.2, PCW 2.1, PML 8.8, PL 3.8, PW 4.6, WL 9.6, FL 3.8, TL 3.8, FA 86.2.

Supplementary Note 4.2 Nymphal sp. 2, exuviae (MGM2016–0.17), (Fig. 1j; Supplementary Figure 13a)

Head and abdomen not preserved; right side of the thorax preserved. Paramedian fissures from the centre to the sides ended laterally at the pronotum; pronotum collar developed. Coxa of all legs elongate and vigorous; trochanter of all legs elbow-like. Profemur massive and slightly bent forward (Supplementary Figure 14a–d); profemur with posterior tooth long and sharp; without an accessory tooth, but with two lateral spines on the outer face of the profemur (Supplementary Figure 14d); one spine at about the middle part of the profemur, the other spine at about the lower part of the profemur, the size of lateral spines close to the size of intermediate; intermediate

tooth robust with a low cusp on distal edge; femoral comb with four teeth, the size of the four teeth similar, the last and the penultimate teeth of femur comb not aggregated (Supplementary Figure 14e). Protibia arched and sickle-shaped; apical tooth long and sharp, sickle-shaped; secondary apical tibial tooth well developed, large and sharp, tooth-like, separated from the apical tooth by a very strong incision, the length of the secondary apical tooth of tibia more than 1/2 the length of the apical tibial tooth. Meso- and metathoracic legs covered with long hairs (Supplementary Figure 15a–c); mesotibia with two rows of spines on both sides, one side with two spines, the other side with three spines (Supplementary Figure 15a, b); the apex of the mesotibia with five distinct spines, one of them longer and dominant, presented as needle-shaped; metatibia with one row of spines on one side, about five spines; the apex of metatibia with five distinct spines, one of them longer and dominant, presented as needle-shaped (Supplementary Figure 15c, d). Tarsus of the hind leg with lateral spines (Supplementary Figure 15d); meso and metapretarsi swollen with a pair of claws of unequal size.

Measurement (mm or degree). WL 3.7, FL 2.9, TL 2.9, FA 70.7.

Supplementary Note 4.3 Nymphal sp. 3, exuviae (LYU–BC2004), (Fig. 1k, Supplementary Figure 13b)

Large compound eye preserved. Antennae filiform and segmented (Supplementary Figure 13e, f). Scape concealed by a ridged prominence on the vertex; pedicel elongate, about twice longer than wide; seven flagellomeres preserved; the length of scape and pedicel slightly longer than the five segments of the flagellum, the diameter of the last two segments tapering. Postclypeus enlarged and swollen, presenting a subobovate shape in ventral view (Supplementary Figure 13d); postclypeus and anteclypeus a depressed deformation covered with hairs; the postclypeus and anteclypeus tightened and narrowed at the location of the connection; the anterior area of the pronotum enclosed by the paramedian fissure, prominent and enlarged, the anterior margin width of this area enclosed by two paramedian fissures as wide as the width of the head. Forewing bud well developed. Foreleg enlarged and

powerful. Coxa brawny and elongate; trochanter elbow-like. Profemur massive and slightly bent forward, with posterior tooth long and sharp; without accessory tooth; with two lateral teeth on the outer face of the profemur (Supplementary Figure 14f, g, i), one spine at about the middle part of the profemur, the other spine at about the lower part of the profemur, the size of lateral spines smaller than the size of intermediate spines; intermediate tooth robust with a low cusp on a distal edge; femoral comb preserved with four teeth (Supplementary Figure 14h), the first tooth relatively independent and projected forward; the last and penultimate tooth of femur comb more aggregated. Protibia arched and like an intrusive scissor; apical tooth very long and sharp; secondary apical tibial tooth well developed, large and sharp, separated from the apical tooth by a very strong incision, the length of the secondary apical tooth of tibia more than 1/2 the length of the apical tibial tooth, blade of tibia developed. One mid or hindleg (probably midleg) preserved independently out of the body (Supplementary Figure 15e), covered with long hairs and echinate ornamentation. Mesotibia with two rows of spines on both sides (Supplementary Figure 15f), one side with two spines, the other side with three spines; the apex of mesotibia with five distinct spines, one of them longer and dominant, presented as needle-shaped. Pretarsi swollen with a pair of unequal size claws.

Measurement (mm or degree). PCL 1.5, PCW 0.69, WL 3.2, FL 1.5, TL 1.4, femoral tooth angle FA 58.4.

Supplementary Note 4.4 Nymphal sp. 4, exuviae (NIGP201900), (Fig. 1l; Supplementary Figure 13c)

Large compound eye preserved. Antenna filiform and segmented (Supplementary Figure 13k); seven segments of flagellum visible, the diameter of the last two segments similar. Rostrum reaching beyond the metacoxae. Two outer mandibular stylets and inner maxillary stylets visible (Supplementary Figure 13h–j); hairy sensilla of the apex of labium visible (Supplementary Figure 13j). Part of the thorax and abdomen structures not visible due to the interference of the amber matrix. Foreleg enlarged and specialized. Coxa of all legs brawny and elongate; trochanter of

all legs elbow-like. Profemur enlarged, powerful and slightly bent forward; posterior tooth long and sharp; accessory tooth and intermediate tooth robust with low cusp on distal edge, accessory tooth blunter than intermediate tooth; femoral comb preserved, with four teeth, the first tooth relatively independent and projected forward; the last and the penultimate tooth of femoral comb more aggregated Supplementary Figure 14k, l, o). Protibia arched, flattened laterally; the secondary apical tooth of tibia relatively small and blunt; the length of the secondary apical tooth of tibia no more than 1/2 the length of the apical tibial tooth; the blade of tibia developed (Supplementary Figure 14k, l). Meso- and metafemora preserved without deformation, slightly bent; trochanter of meso- and metalegs elbow-like. Midleg and hindleg covered with hairs (Supplementary Figure 15g–i); the part of meso- and metatibiae close to femur, slightly bent and shrunk; the apex spines of meso- and metatibiae preserved, incomplete; the apex of left mesotibia preserved with three spines, one of which is the dominant and longer spine; the apex of right mesotibia preserved with one dominant and longer spine; the apex of right metatibia preserved with three spines with one dominant and longer spine; the dominant spine presented as needle-shaped. Tarsus of all legs with two segments; pretarsi with unequal size claws but not swollen (Supplementary Figure 15g–i, p).

Measurement (mm or degree). BL 9.6, FL 2.0, TL 1.8, FA 63.8.

Supplementary Note 4.5 Nymphal sp. 5, exuviae (NIGP201901), (Fig. 1m)

Head incomplete, crown preserved with compound eyes. The anterior area of the pronotum enclosed by prominent paramedian fissure, the anterior margin width of this area enclosed by two paramedian fissures slightly shorter than the width of the head. Right foreleg preserved (Supplementary Figure 14m, n). Coxa robust; trochanter elbow-like. Profemur massive and slightly bent forward; posterior tooth long and sharp; accessory tooth and intermediate tooth sharp, accessory tooth slightly smaller than intermediate tooth; femoral comb preserved with four teeth (Supplementary Figure 14p), the anterior tooth relatively independent and projecting forward; the size

of the four teeth similar, the last and the penultimate tooth of femur comb not aggregated. Abdomen elongated and not complete. Gender indistinguishable.

Measurement (mm or degree). BL 12.9, FL 3.0, TL 3.7, FA 70.

Supplementary Note 5. Description for the scheme outlining the forewing venation of Cicadoidea in this study

Studying insect wing venation is important for understanding the morphological variations and diversity of modern and extinct species (Shimmi et al., 2014; Breitkreuz et al., 2017). Although the vein pattern is related to the evolution of insects, there are still many uncertainties about the homology of veins among different insects. The determination of the name and homology of the wing vein is currently based on a variety of features of analysis, including trachea, hemolymph, nerve, fossil inference, and others (Comstock and Needham, 1898; Dworakowska, 1988; Kukalová-Peck, 1991; Hamilton et al., 1971). However, differing results are still controversial and need to be verified considering the complexity and difficulty of the fusion and loss of wing veins (Nel, A. et al., 2012). Based on the research results of the most commonly used data on tracheation from nymphs and the observation of the research materials in this paper (Comstock and Needham, 1898), MA and MP veins are used after the primary separation of M veins for the principle of the technical unity of terms. The terms related to M vein are used in this article without regard to homology comparison with other taxa. Their homology will be discussed in other papers. Additionally, we discovered the short RA_1 vein of the forewing near the nodal line in extant cicadas (Supplementary Figure 19). This vein had not been observed in adult studies before[86], but it is clearly observed in our fossils, and was supported by the development of the vein trachea in the young nymph (Comstock and Needham, 1898). From further morphological observation of the wings of modern adult cicadas, we believe that there is a feature relatively close to the nodal line R_1 vein in Cicadoidea, which is not apparent but expressed.

Supplementary Figures

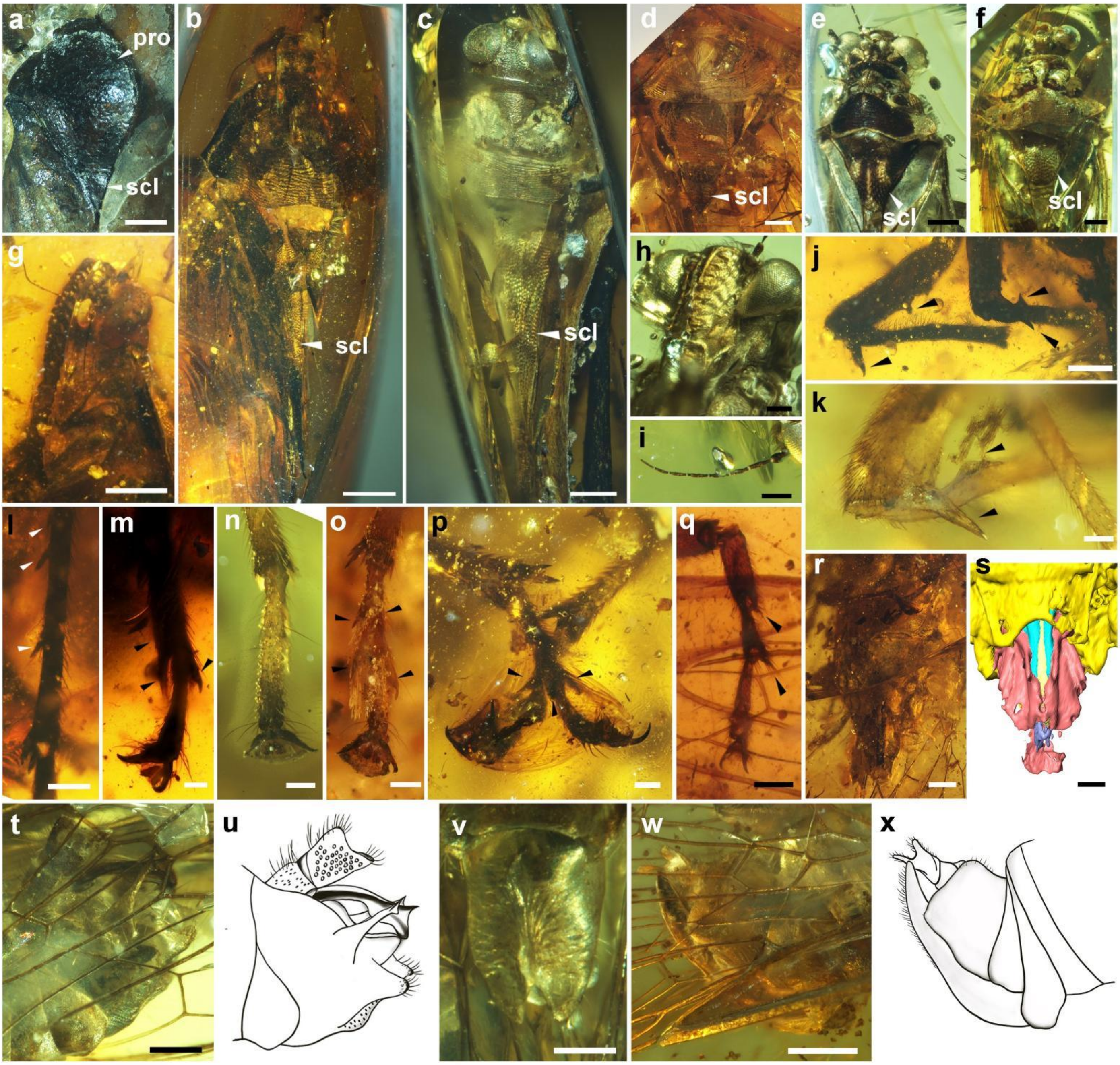

Supplementary Figure 1. Details of head, notum, leg, and genitalic structures from adult cicadoid fossils. a–**f**, Dorsal view of the head and notum of the examined fossils. **a**, *Eunotalia emeryi*. gen. et sp. nov. **b**, *Cretotettigarcta problematica* comb. nov. **c**, *C. shcherbakovi* sp. nov. **d**, *Vetuprosbole parallelica*. **e**, *Pranwanna xiai* gen. et sp. nov, male. **f**, *Pr. xiai,* female. **g**, Left view of the head of *C. problematica*. **h**, ventral view of the head of *Pr. xiai*. **i**, Lateral view of antenna of *Pr. xiai*, showing

one pedicel and seven flagella segments. **j**, **k**, Forelegs of *C. problematica* and *C. shcherbakovi*; arrows showing foreleg spines. **l**, Metatibia of *C. problematica*; arrows showing three lateral, metatibial spines. **m**, Metatarsi of *C. problematica*; arrows showing two pairs of metatarsal lateral spines. **n**, Protarsi of *C. shcherbakovi*. **o**, Mesotarsi of *C. shcherbakovi*, showing two pairs lateral, mesotarsal spines. **p**, Metatarsi of *V. parallelica*. **q**, Metatarsi of *Pr. xiai* from paratype specimen, arrows showing metatarsi first two segments have lateral spines and with dense setae. **r**–**s**, Ventral view of genitalia of *V. parallelica*. **t**–**v**, Male genitalia of *Pr. xiai*. **t**, Left view of genitalia, illustrated in light micrographs. **u**, Overlay drawing from (t). **v**, Rear view of genitalia in (t). **w**, Left view of female genitalia of *Pr. xiai* gen. et sp. nov., illustrated in light micrographs. **x**, Overlay drawing of left view from (w). scl, scutellum.

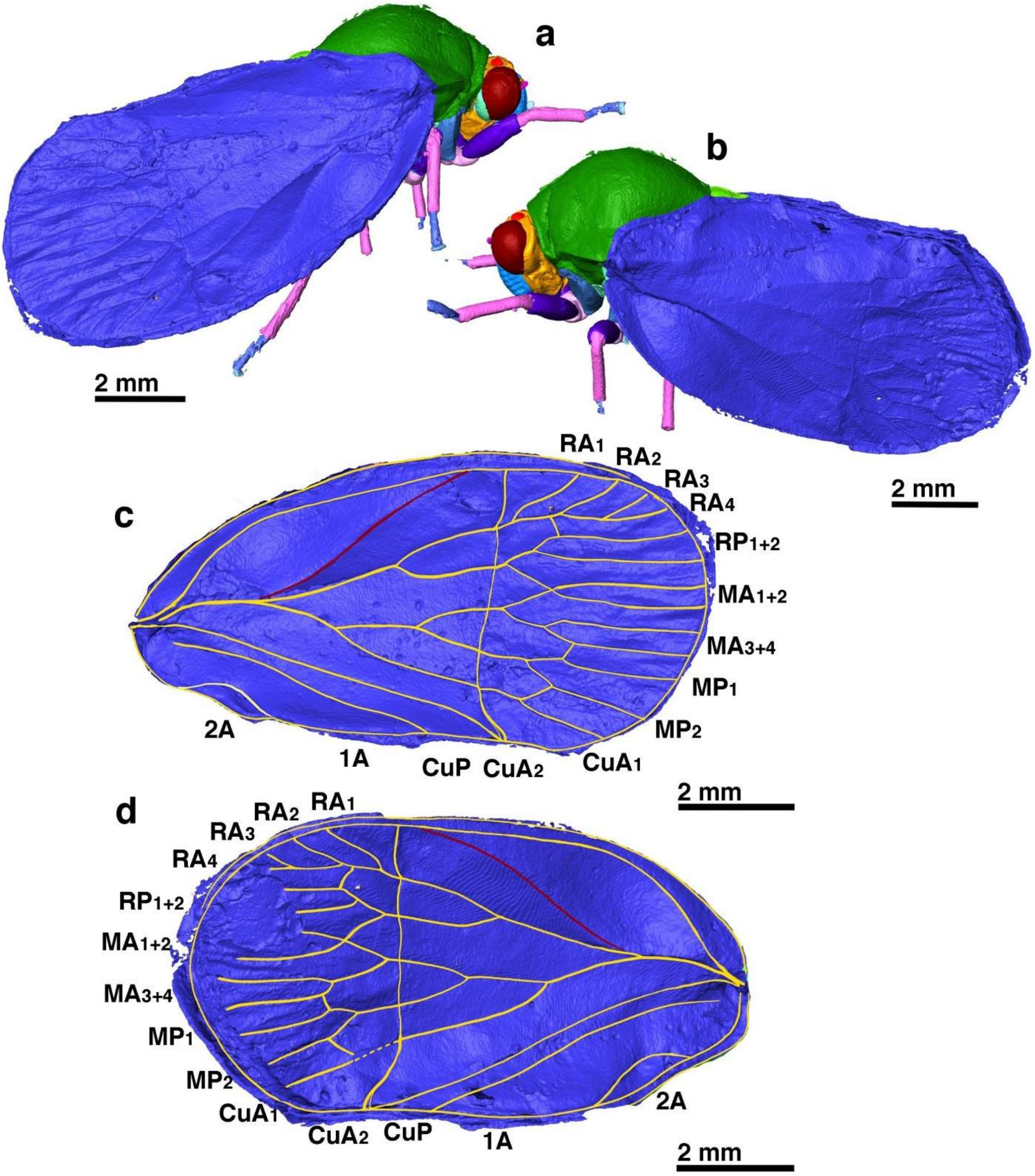

Supplementary Figure 2. Microtomographic reconstruction of *Eunotalia emeryi* gen. et sp. nov. (MGM2016–014). a, Right view. **b**, Left view. **c**, Right forewing

with overlay drawing of venation. **d**, Left forewing with overlay drawing of venation. Colour coding of non-wing structures as in Fig. 2. Each colour in the figure represents a different structure, and these colour-structure associations remain consistent throughout the figure even when the label is not repeated in each panel. The forewings are represented in blue and wing veins are represented in yellow.

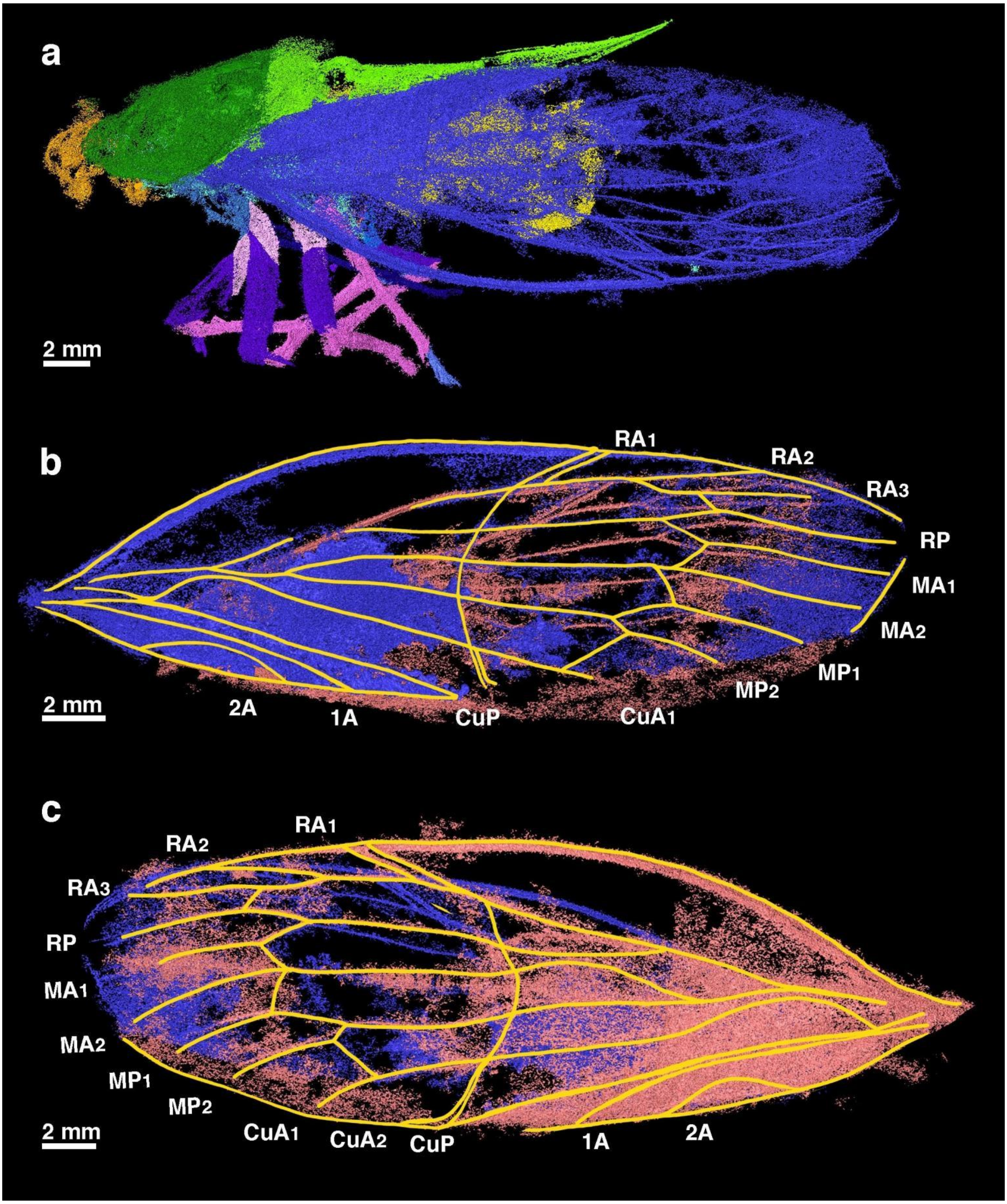

Supplementary Figure 3. Microtomographic reconstruction of *Cretotettigarcta problematica* comb. nov. (New material: NIGP201895). a, Left view of the entire body. **b**, Right forewing with overlay drawing of venation. **c**, Left hindwing with overlay drawing of venation. Colour coding of non-wing structures as in Fig. 2. Each

colour in the figure represents a different structure, and these colour-structure associations remain consistent throughout the figure even when the label is not repeated in each panel. The right wing is represented in blue, the left wing in pink, and wing veins are represented in yellow.

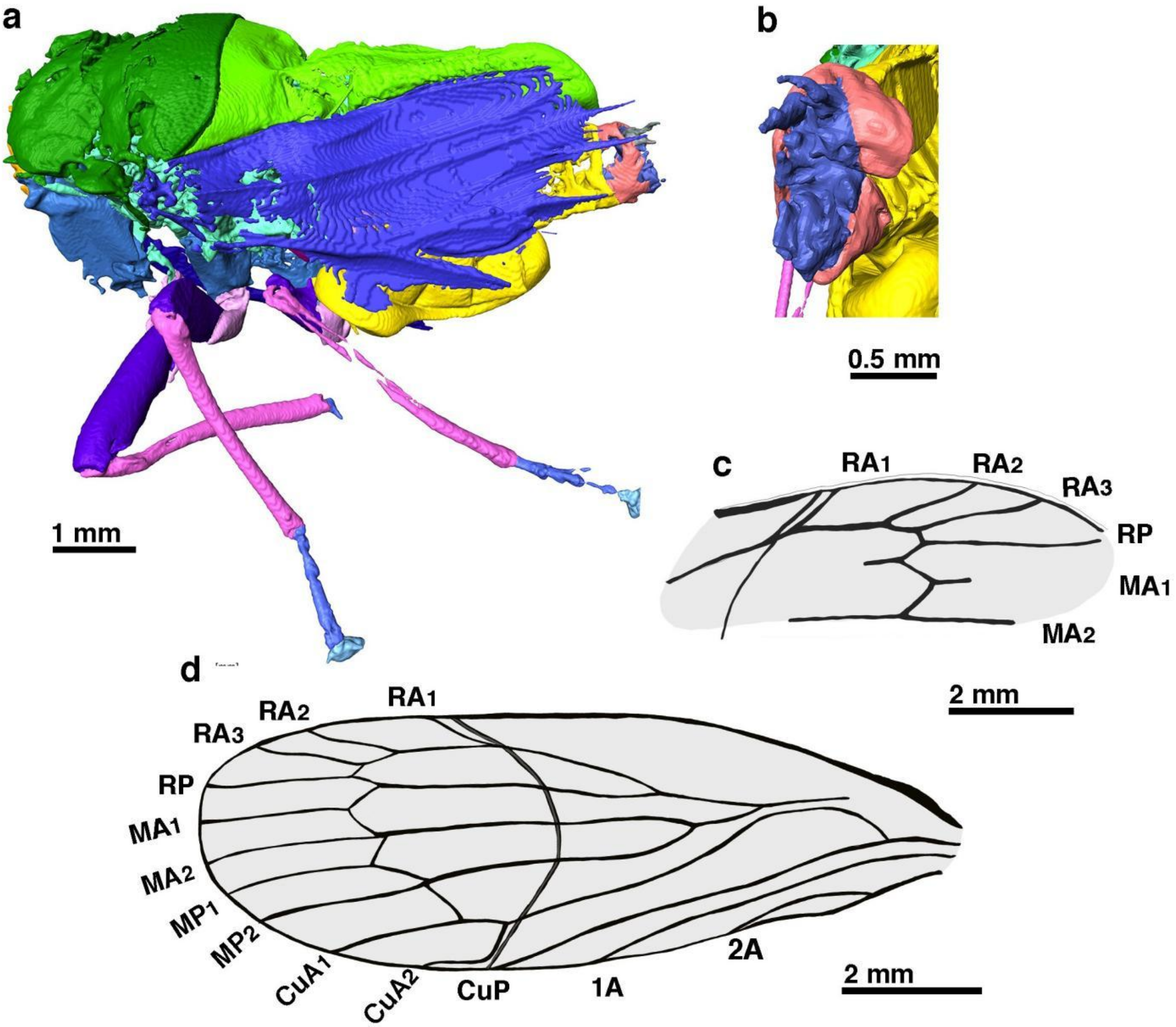

Supplementary Figure 4. ***Cretotettigarcta shcherbakovi*** **sp. nov. (NIGP201896). a**, Left view of a microtomographic reconstruction, showing part of forewing venation that is vaguely observed under the light microscope. Colour coding of non-wing structures as in Fig. 2. Each colour in the figure represents a different structure, and these colour-structure associations remain consistent throughout the figure even when the label is not repeated in each panel. **b**, Male genitalia from a micro-CT scan in blue. **c**, Overlay drawing of the left forewing. **d**, Overlay drawing of a portion of the right forewing. The membrane of the wings is shown in grey.

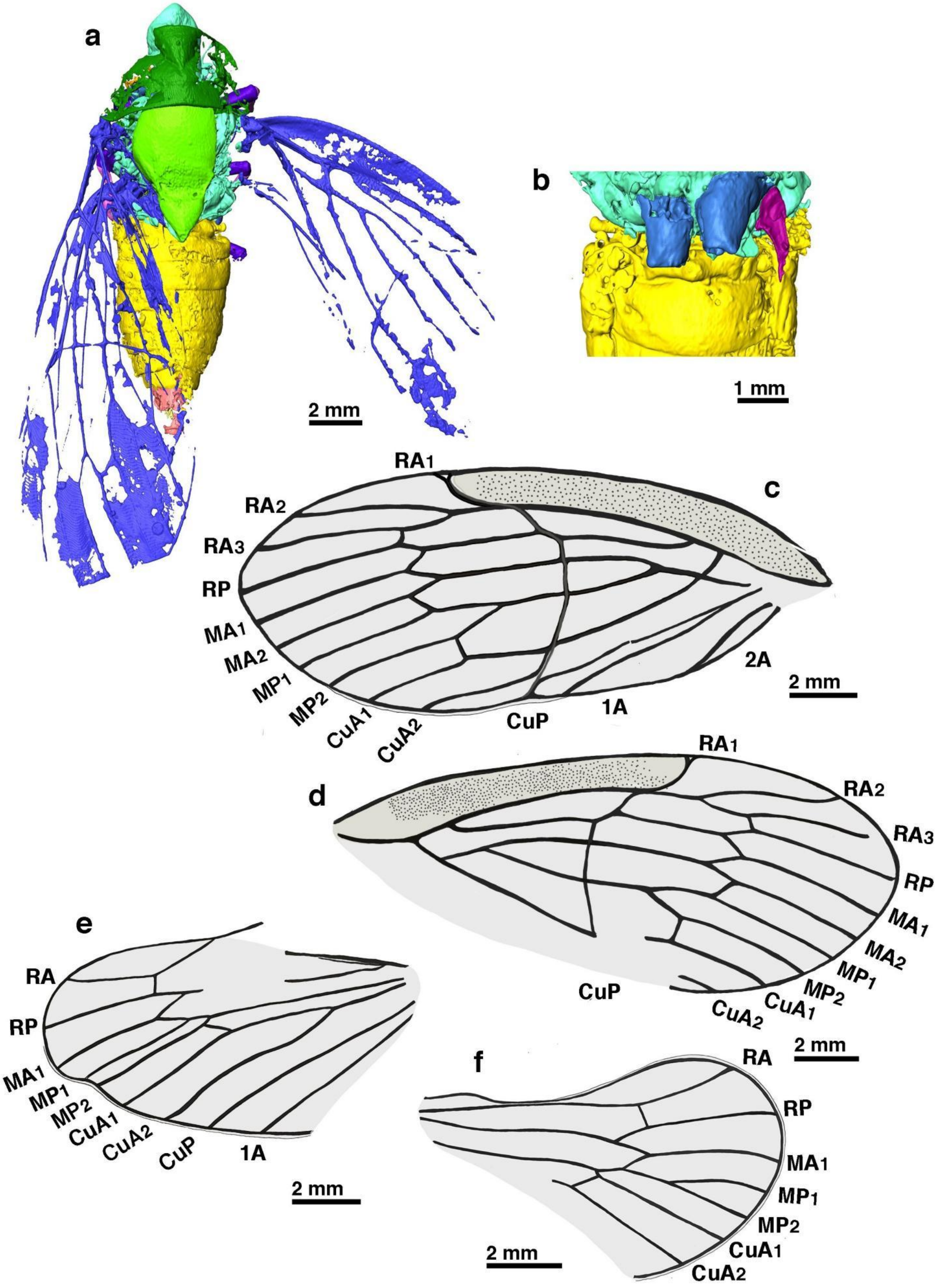
a
2 mm
b
1 mm
c
RA1
RA2
RA3
RP
MA1
MA2
MP1
MP2
CuA1
CuA2
CuP
1A
2A
2 mm
d
RA1
RA2
RA3
RP
MA1
MA2
MP1
MP2
CuA1
CuA2
CuP
2 mm
e
RA
RP
MA1
MP1
MP2
CuA1
CuA2
CuP
1A
2 mm
f
RA
RP
MA1
MP1
MP2
CuA1
CuA2
2 mm

Supplementary Figure 5. New specimen of the *Vetuprosbole parallelica*. (New material: NIGP201897). a, Dorsal view of microtomographic reconstruction, showing the wings. **b**, Ventral view, showing the meracanthus extending beyond the second segment of the abdomen. **c**, Overlay drawing of left forewing. **d**, Overlay drawing of right forewing. **e**, Overlay drawing of a portion of the left hindwing. **f**, Overlay drawing of a portion of the right hindwing. Colour coding of non-wing structures as in Fig. 2. Each colour in the figure represents a different structure, and these colour-structure associations remain consistent throughout the figure even when the label is not repeated in each panel. The membrane of the wings is shown in grey.

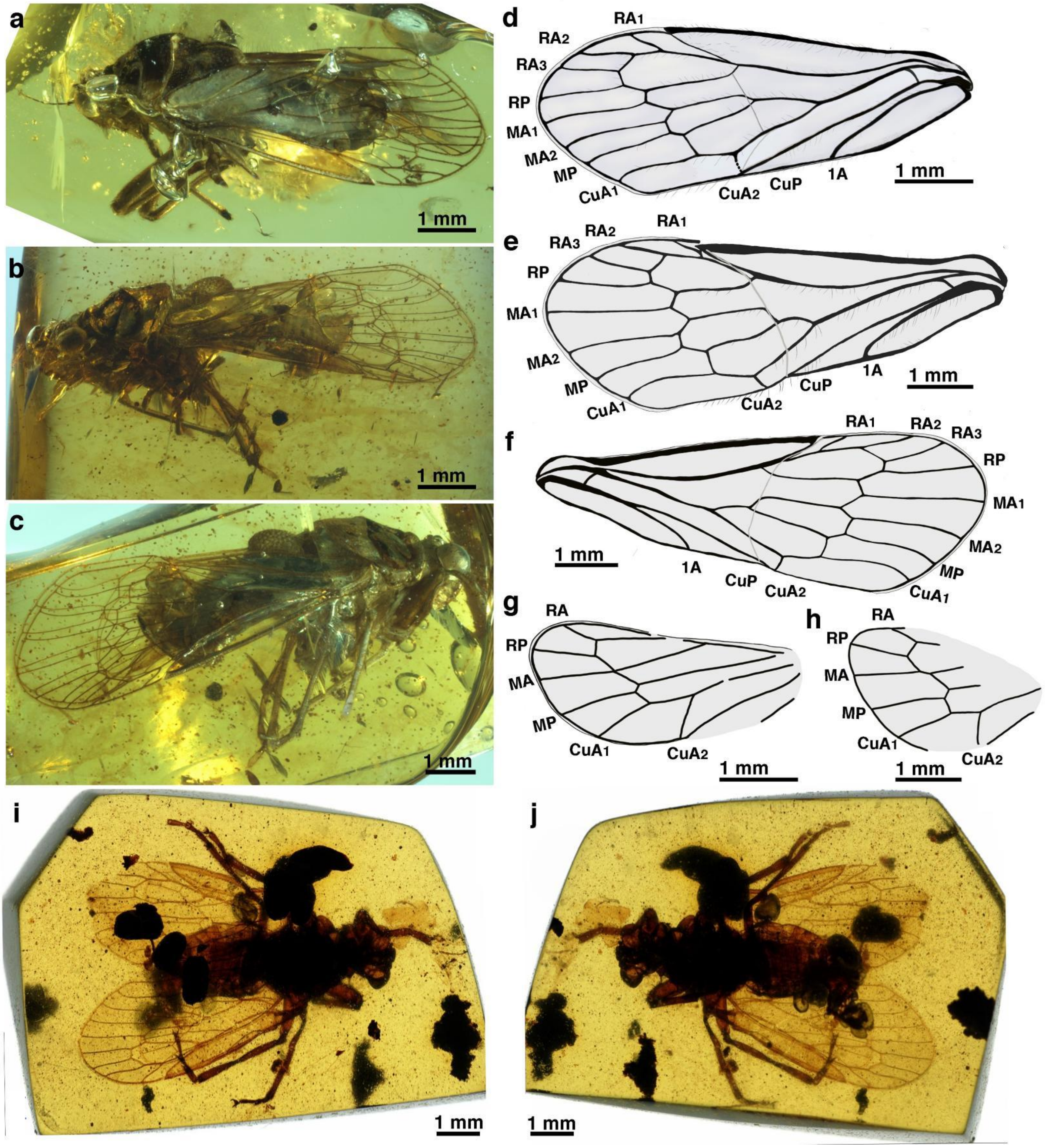
a
1 mm
b
1 mm
c
1 mm
d
RA1
RA2
RA3
RP
MA1
MA2
MP
CuA1
CuA2
CuP
1A
1 mm
e
RA3
RA2
RA1
RP
MA1
MA2
MP
CuA1
CuA2
CuP
1A
1 mm
f
RA1
RA2
RA3
RP
MA1
MA2
MP
CuA1
CuA2
CuP
1A
1 mm
g
RA
RP
MA
MP
CuA1
CuA2
1 mm
h
RA
RP
MA
MP
CuA1
CuA2
1 mm
i
1 mm
j
1 mm

Supplementary Figure 6. ***Pranwanna xiai*** **gen. et sp. nov. a**, Holotype LYU–BC2001, male Specimen. **b**, **c**, Allotype LYU–BC2002, female specimen. **b**, Left lateral view. **c**, Right lateral view. **d–f**, Overlay drawings of forewings. **d**, Overlay drawings of left forewing from (a). **e**, Overlay drawings of left forewing from (b). **f**, Overlay drawings of right forewing from (c). **g**, Overlay drawings of left hindwing from (a) specimen. **h**, Overlay drawings of left hindwing from (b). **i**, **j**, Paratype of male specimen (LYU–BC2004). **i**, Dorsal view. **j**, Ventral view. The membrane of the wings is shown in grey.

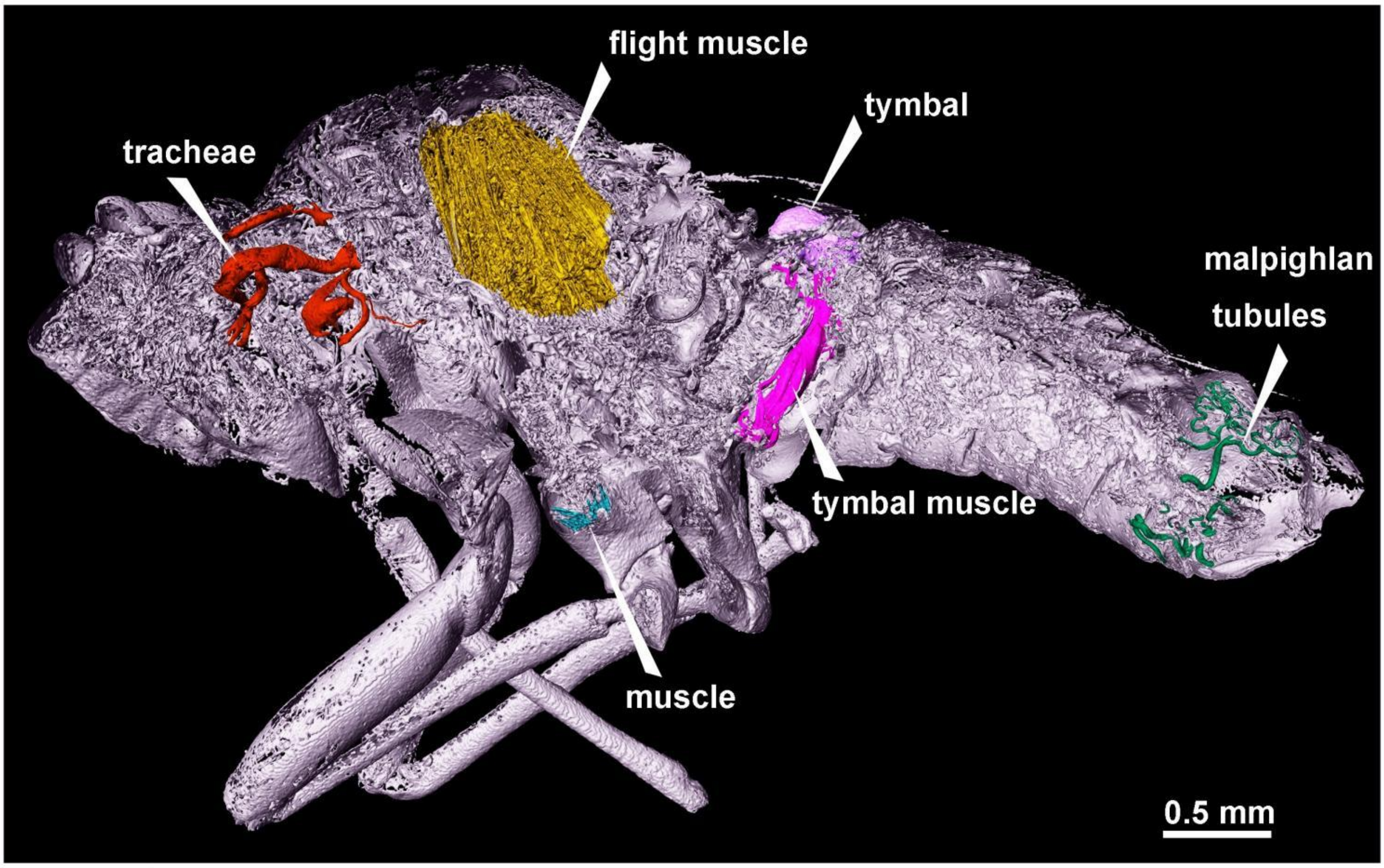

Supplementary Figure 7. Micro-CT reconstruction along the sagittal section of *Pranwanna xiai* gen. et sp. nov., showing internal body structures.

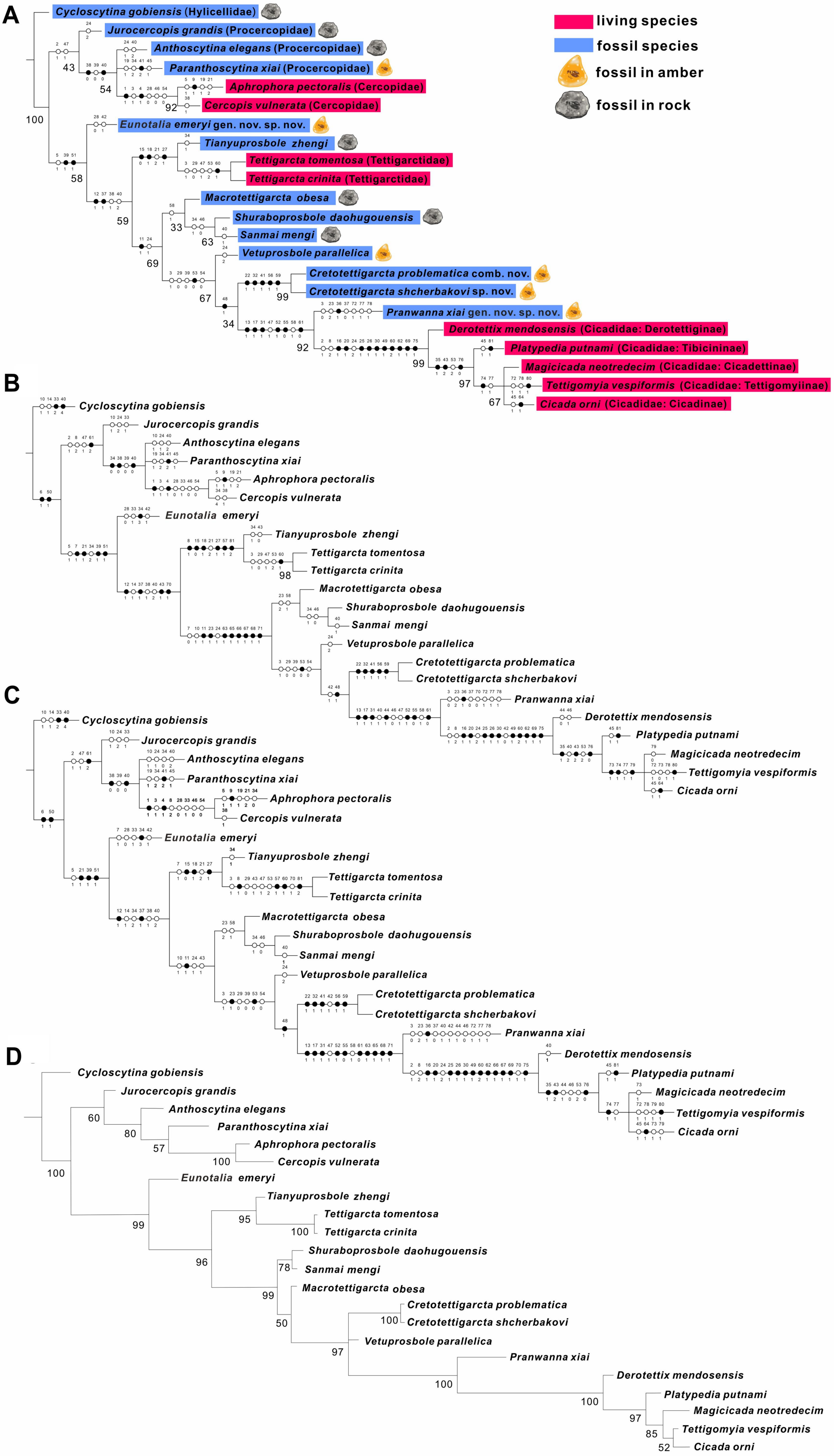
A
living species
fossil species
fossil in amber
fossil in rock
Cycloscytina gobiensis (Hylicellidae)
Jurocercopis grandis (Procercopidae)
Anthoscytina elegans (Procercopidae)
Paranthoscytina xiai (Procercopidae)
Aphrophora pectoralis (Cercopidae)
Cercopis vulnerata (Cercopidae)
Eunotalia emeryi gen. nov. sp. nov.
Tianyuprosbole zhengi
Tettigarcta tomentosa (Tettigarctidae)
Tettigarcta crinita (Tettigarctidae)
Macrotettigarcta obesa
Shuraboprosbole daohugouensis
Sanmai mengi
Vetuprosbole parallelica
Cretotettigarcta problematica comb. nov.
Cretotettigarcta shcherbakovi sp. nov.
Pranwanna xiai gen. nov. sp. nov.
Derotettix mendosensis (Cicadidae: Derotettiginae)
Platypedia putnami (Cicadidae: Tibicininae)
Magicicada neotredecim (Cicadidae: Cicadettinae)
Tettigomyia vespiformis (Cicadidae: Tettigomyiinae)
Cicada orni (Cicadidae: Cicadinae)
B
Cycloscytina gobiensis
Jurocercopis grandis
Anthoscytina elegans
Paranthoscytina xiai
Aphrophora pectoralis
Cercopis vulnerata
Eunotalia emeryi
Tianyuprosbole zhengi
Tettigarcta tomentosa
Tettigarcta crinita
Macrotettigarcta obesa
Shuraboprosbole daohugouensis
Sanmai mengi
Vetuprosbole parallelica
Cretotettigarcta problematica
Cretotettigarcta shcherbakovi
Pranwanna xiai
Derotettix mendosensis
Platypedia putnami
Magicicada neotredecim
Tettigomyia vespiformis
Cicada orni
C
Cycloscytina gobiensis
Jurocercopis grandis
Anthoscytina elegans
Paranthoscytina xiai
Aphrophora pectoralis
Cercopis vulnerata
Eunotalia emeryi
Tianyuprosbole zhengi
Tettigarcta tomentosa
Tettigarcta crinita
Macrotettigarcta obesa
Shuraboprosbole daohugouensis
Sanmai mengi
Vetuprosbole parallelica
Cretotettigarcta problematica
Cretotettigarcta shcherbakovi
Pranwanna xiai
Derotettix mendosensis
Platypedia putnami
Magicicada neotredecim
Tettigomyia vespiformis
Cicada orni
D
Cycloscytina gobiensis
Jurocercopis grandis
Anthoscytina elegans
Paranthoscytina xiai
Aphrophora pectoralis
Cercopis vulnerata
Eunotalia emeryi
Tianyuprosbole zhengi
Tettigarcta tomentosa
Tettigarcta crinita
Shuraboprosbole daohugouensis
Sanmai mengi
Macrotettigarcta obesa
Cretotettigarcta problematica
Cretotettigarcta shcherbakovi
Vetuprosbole parallelica
Pranwanna xiai
Derotettix mendosensis
Platypedia putnami
Magicicada neotredecim
Tettigomyia vespiformis
Cicada orni

Supplementary Figure 8. For complete cladograms resulting from the phylogenetic analyses. a–c, Cladograms obtained from the analysis of TNT (v.1.6) and displayed by Winclada 1.00.08. Bootstrap values at the nodes. Filled circles represent unique changes, open circles represent non-unique changes. **a**, Using equal weighting, unambig changes only result of the strict consensus tree. **b**, Using equal weighting, slow optimization (DELTRAN) result of the strict consensus tree. **c**, Using equal weighting, fast optimization (ACCTRAN) result of the strict consensus tree. **d**, The tree obtained from the analysis of MrBayes (v.3.2.7a). Percent posterior probabilities are below the branches. For characters description, matrix, and additional explanatory data, see Materials and Methods, Supplementary note 2, and Supplementary Table 2.

Here, we present the multiple results of parsimony ancestral character reconstructions of the strict consensus tree conducted under the condition of using equal weighting (Supplementary Fig. 8a, b and c). Due to the different methods used, there will be ambiguities in the placement of certain parsimony ancestral character reconstructions on the phylogenetic tree. However, the differences in the positions of these reconstructed features on the phylogenetic tree branches do not affect the discussions and conclusions of this paper. Further judgment on the placement of these reconstructed features requires more evidence. In this paper, we initially list these possibilities for consideration.

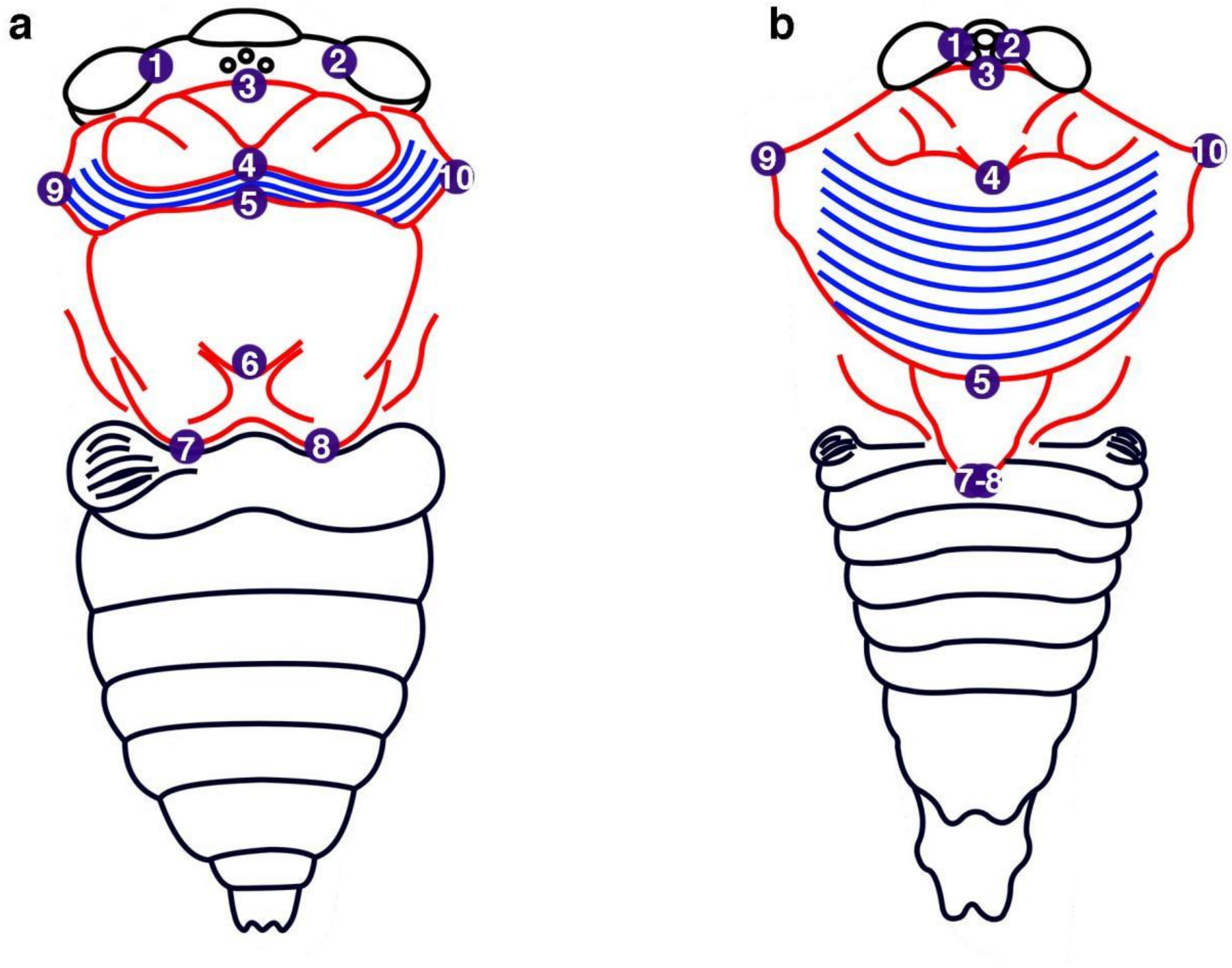

Supplementary Figure 9. Landmarks modeling of the dorsal profile of the Cicadoidea head and notum. Shown are eight landmarks associated eight characteristics from dorsal profile of the notum and head. **a**, Landmarks shown in a schematic of the line drawings in a modern Cicadidae. **b**, Landmarks shown in a schematic of the line drawing in a modern Tettigarctidae. Line drawings referenced from Moulds, 1990 and Zhou and Lei, 1997. Red lines depict the thorax in dorsal view, blue lines depict the wrinkled pronotal collar structure of the pronotum, and black lines depict the head and abdomen in dorsal view.

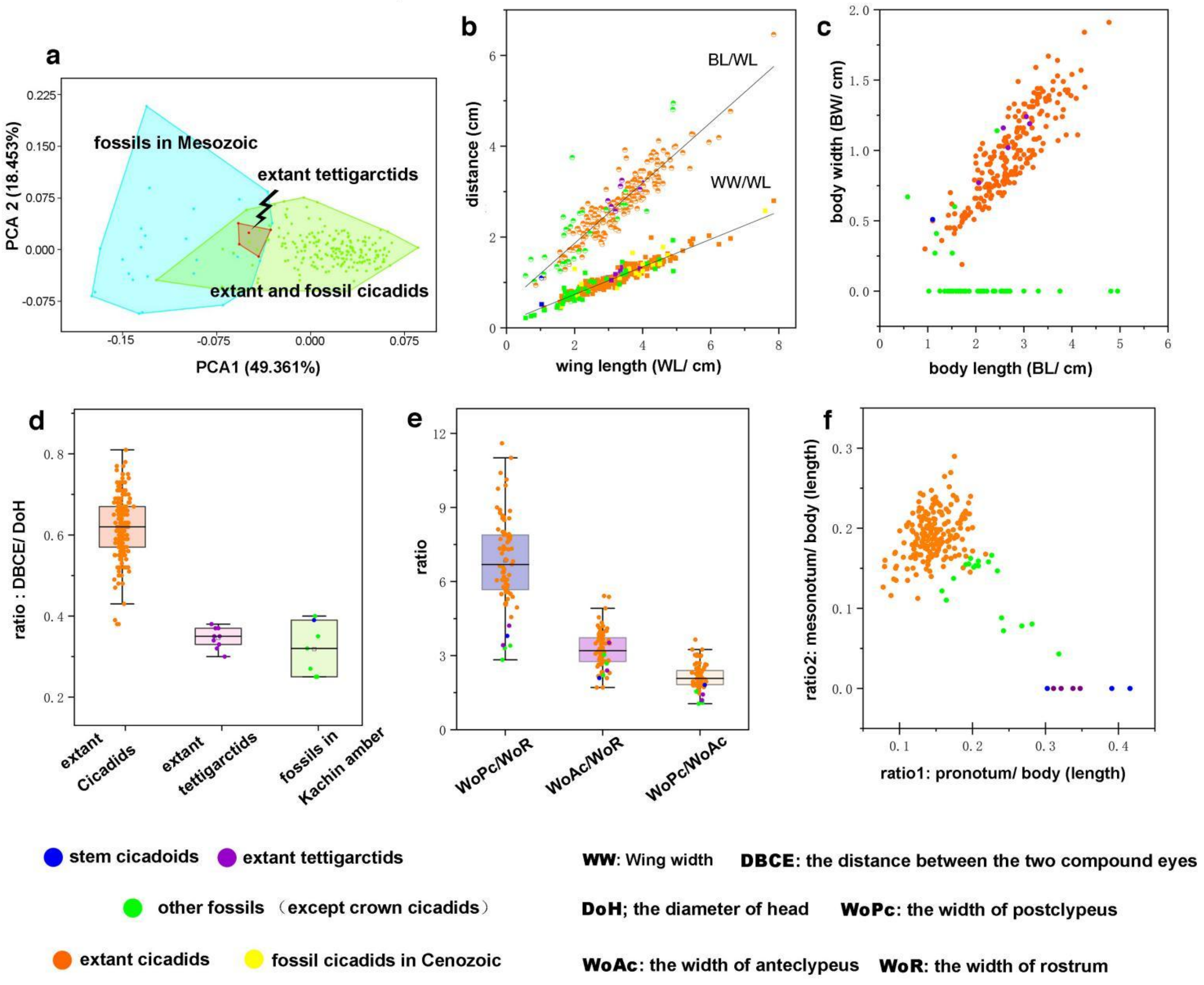

Supplementary Figure 10. Morphometric measurement analyses data of adults. a, Ordination plot of geometric morphometrics (GM) analysis of the forewing profile. **b**, Scatter plots of the body length versus wing length, and wing width versus wing length. **c**, Scatter plots of the body length versus body width. **d**, Boxplot showing the ratio of the distance between the two compound eyes and adult head diameter. **e**, Boxplot showing the ratios of the width of the postclypeus versus the width of the rostrum, the width of the anteclypeus versus the width of the rostrum, and the width of the postclypeus versus the width of the anteclypeus for adults. **f**, Scatter plots of the length of mesonotum/body versus the length of pronotum/body. Abbreviations are at the bottom of the diagram, colored dots are descriptions for b–f. For access to data used in this Figure, please see the Source Data file.

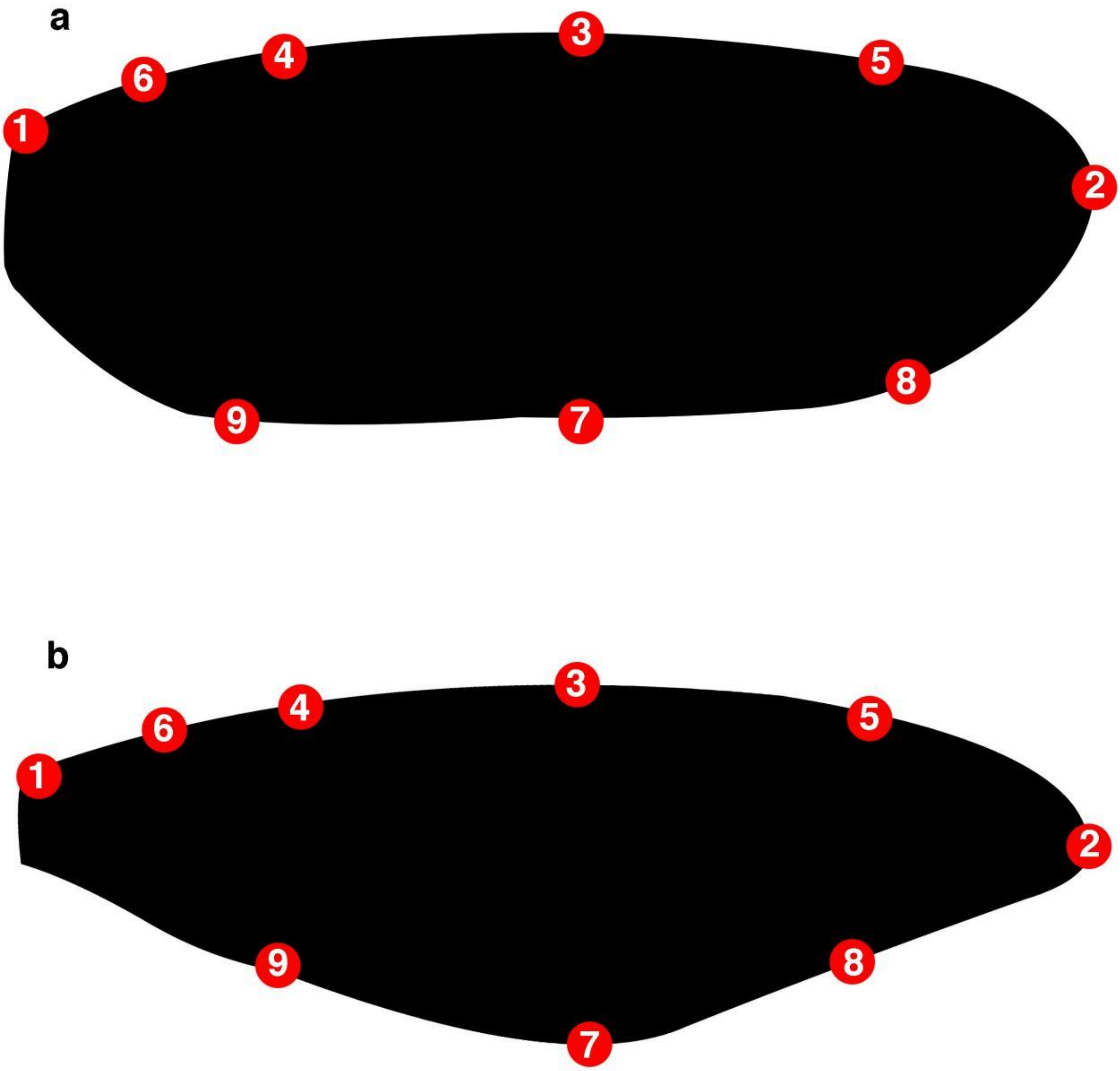

Supplementary Figure 11. Landmarks modeling of the Cicadoidea forewing. Shown are nine landmarks associated with nine characteristics from forewing profile. **a**, A forewing of *Mesodiphthera grandis* Tillyard, 1919 from the Triassic for an example. **b**, A forewing of modern *Thopha saccata* (Fabricius, 1803). For the definitions of each landmark, please refer to Supplementary Table 6.

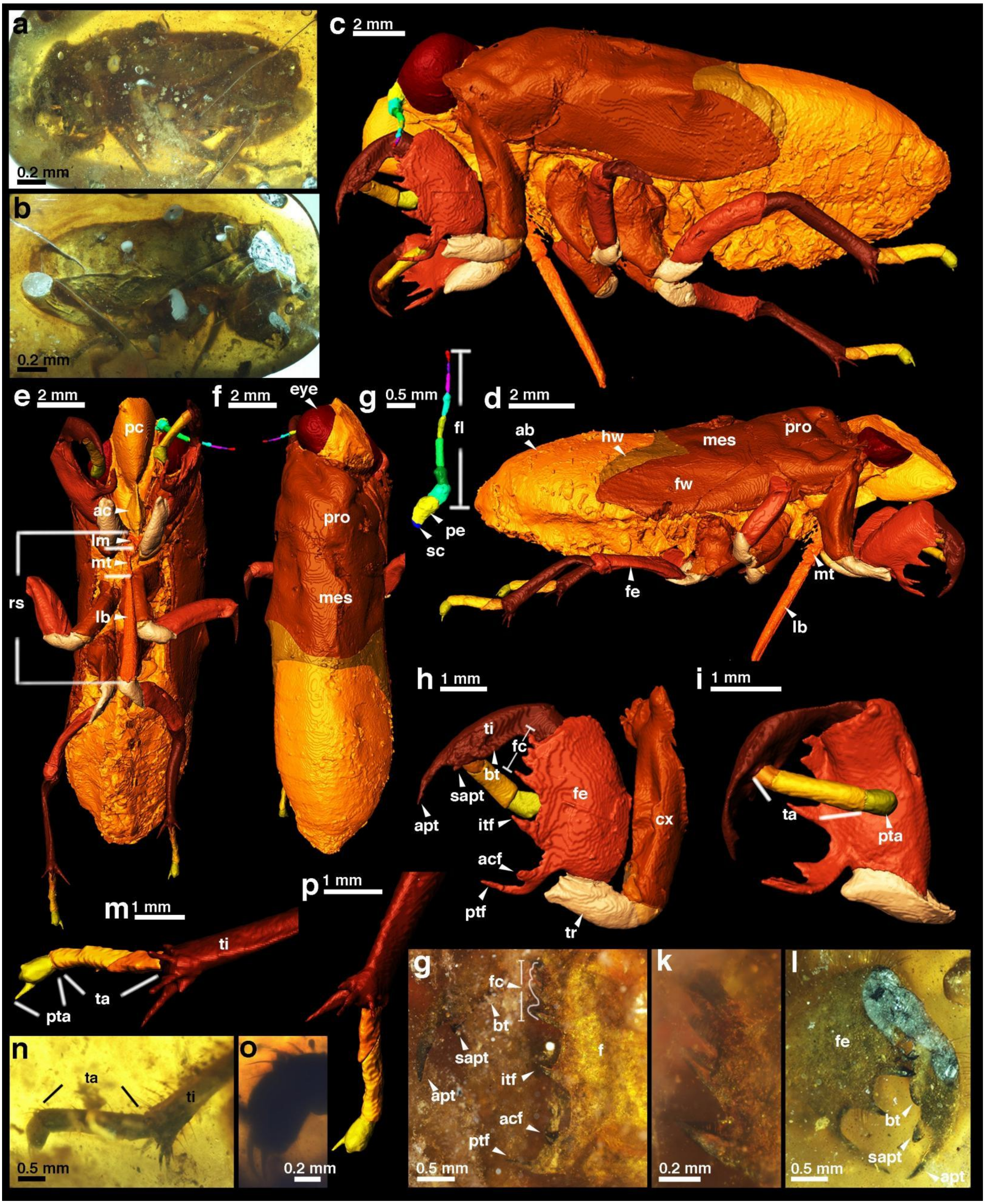
a
0.2 mm
b
0.2 mm
c 2 mm
e 2 mm
pc
ac
lm
mt
rs
lb
f 2 mm
eye
pro
mes
g 0.5 mm
fl
pe
sc
d 2 mm
ab
hw
mes
pro
fw
fe
mt
lb
h 1 mm
ti
fc
bt
sapt
apt
itf
fe
cx
acf
ptf
tr
i 1 mm
ta
pta
m 1 mm
ti
ta
pta
p 1 mm
g
fc
bt
sapt
apt
itf
f
acf
ptf
0.5 mm
k
0.2 mm
l
fe
bt
sapt
apt
0.5 mm
n
ta
ti
0.5 mm
o
0.2 mm

Supplementary Figure 12. Final-instar nymph of Cicadoidea in Kachin amber. Cicadoidea nymphal sp. 1 (NIGP201898), reflected in light micrographs (a, b, n–o, j–l) and in microtomographic reconstructions (c–i, m, p). **a**–**f**, Habitus. **a**, Left view. **b**, Right view. **c**, Left view. **d**, Right view. **e**, Ventral view. **f**, Dorsal view. **g**, Left antenna. **h**, Left foreleg. **i**, Right foreleg. **j**, Left profemur and tibia. **k**, Femoral comb. **l**, Right profemur and tibia. **m**–**n**, Right hind leg. **o**, Pretarsi of right hind leg. **p**, Left hind leg. Abbreviations: ab, abdomen; ac, anteclypeus; acf, accessory tooth of femur; apt, apical tooth of tibia; bt, blade of tibia; clw, pretarsal claw; cox, coxa; e, compound eye; fc, femoral comb; fem, femur; fw, forewing bud; hw, hindwing bud; itf, intermediate tooth of femur; labi, labium; labr, labrum; m, mentum; mes, mesonotum; sapt, secondary apical tooth of tibia; pc, postclypeus; pe, pedicel; pro, pronotum; pta, pretarsi; ptf, posterior tooth of femur; r, rostrum; scp, scape; tar, tarsus; tib, tibia; and tro, trochanter. The colours in the figure represents a different structure, and these colour-structure associations remain consistent throughout the figure even when the label is not repeated in each panel.

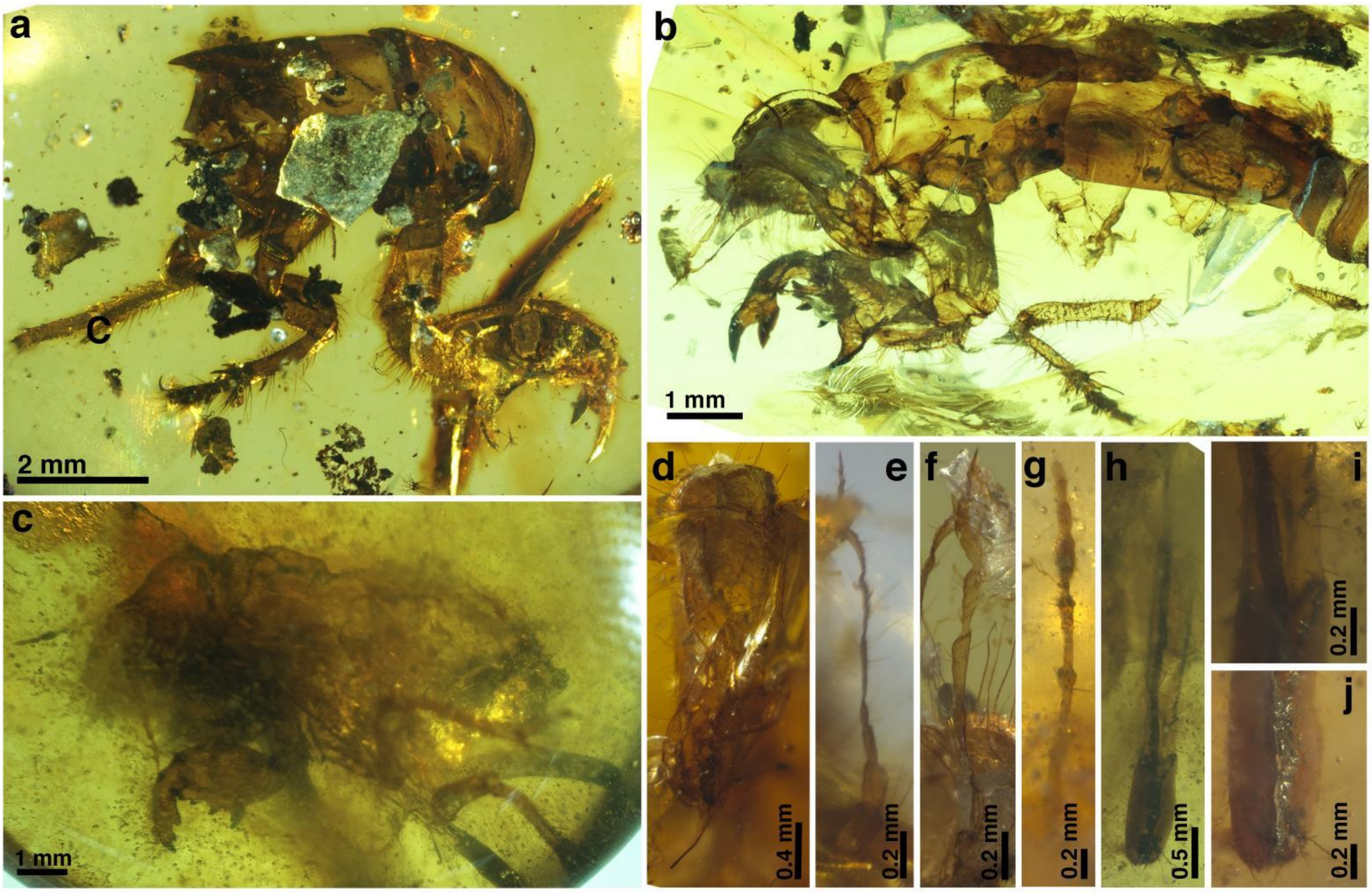

Supplementary Figure 13. Final-instar exuviae of Cicadoidea in Kachin amber. a, Nymphal sp. 2 (MGM2016–0.17), right view. **b**, Nymphal sp. 3 (LYU–BC2004), left view. **c**, Nymphal sp. 4 (NIGP201900), left view. **d**, Postclypeus of sp. 2. **e**, Right antenna of sp. 3. **f**, Left antenna of sp. 3. **g**, Left antenna of sp. 4. **h–j**, Rostrum of sp. 4. **i**, Enlarged from (h), showing part of the maxillary stylets. **j**, Enlarged from (h), showing hairy sensilla of the apex of the labium.

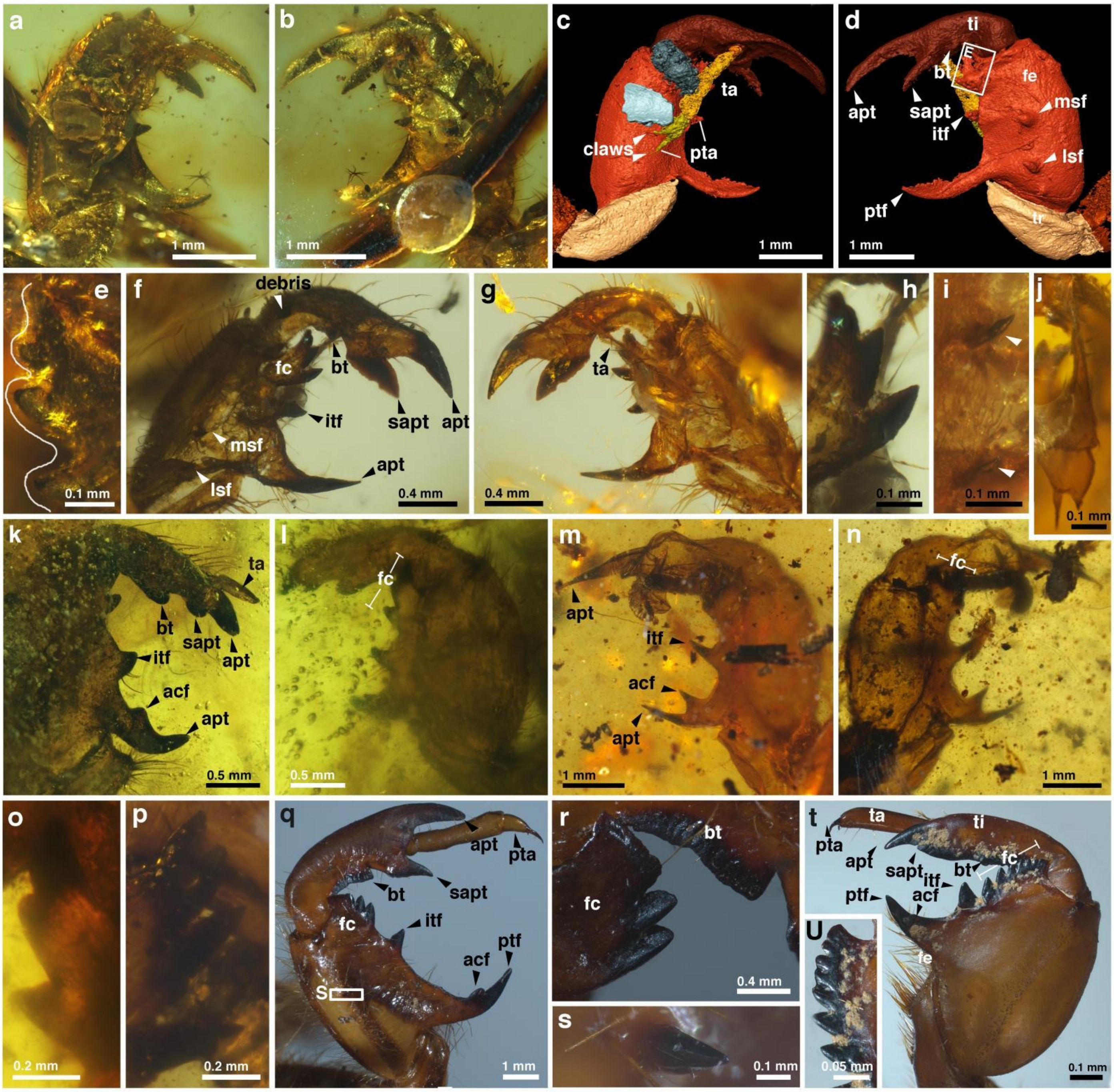

Supplementary Figure 14. Comparisons of forelegs of final-instar cicadoid exuviae from Kachin amber and extant species. Forelegs of final-instar cicadoid exuviae from Kachin amber (a–p) and extant final-instar nymphal exuviae (q–u), illustrated in light micrographs (a, b, e–t) and microtomographic reconstructions (c, d). **a**–**e**, Foreleg of nymphal sp. 2 (MGM2016–0.17). **a**, Right view. **b**, Left view. **c**, Right view. **d**, Left view. **e**, Femoral comb. **f**–**j**, Foreleg of nymphal sp. 3 (LYU–BC2004). **f**,

Right view. **g**, Left view. **h**, Femoral comb. **i**, Two lateral spines on the outer femoral surface. **j**, Fore tarsus and pretarsi. **k–l**, Foreleg of nymphal sp. 4 (NIGP201900). **k**, Right foreleg in right view. **l**, Left foreleg in left view. **m–n**, Foreleg of sp. 5 in left view and right view, respectively. **o**, Femoral comb of nymphal sp. 5 (NIGP201901). **p**, Femoral comb of sp. 5. **q–s**, Right foreleg of *Tettigarcta tomentosa* (extant Tettigarctidae) in right view. **q**, Habitus. **r**, Femoral comb and blade of tibia. **s**, Lateral spine shown by white square in (q). **t–u**, Left foreleg of *Hyalessa maculaticollis* (extant Cicadidae) in left view. **t**, Habitus. **u**, Femoral comb. Abbreviations: acf, accessory tooth of femur; apt, apical tooth of tibia; bt, blade of tibia; clw, pretarsal claw; cox, coxa; fc, femoral comb; fem, femur; itf, intermediate tooth of femur; lsf, lower lateral spine on the outer surface of femur; msf, mid lateral spine on the outer surface of femur; sapt, secondary apical tooth of tibia; pta, pretarsi; ptf, posterior tooth of femur; tar, tarsus; tib, tibia; tro, trochanter.

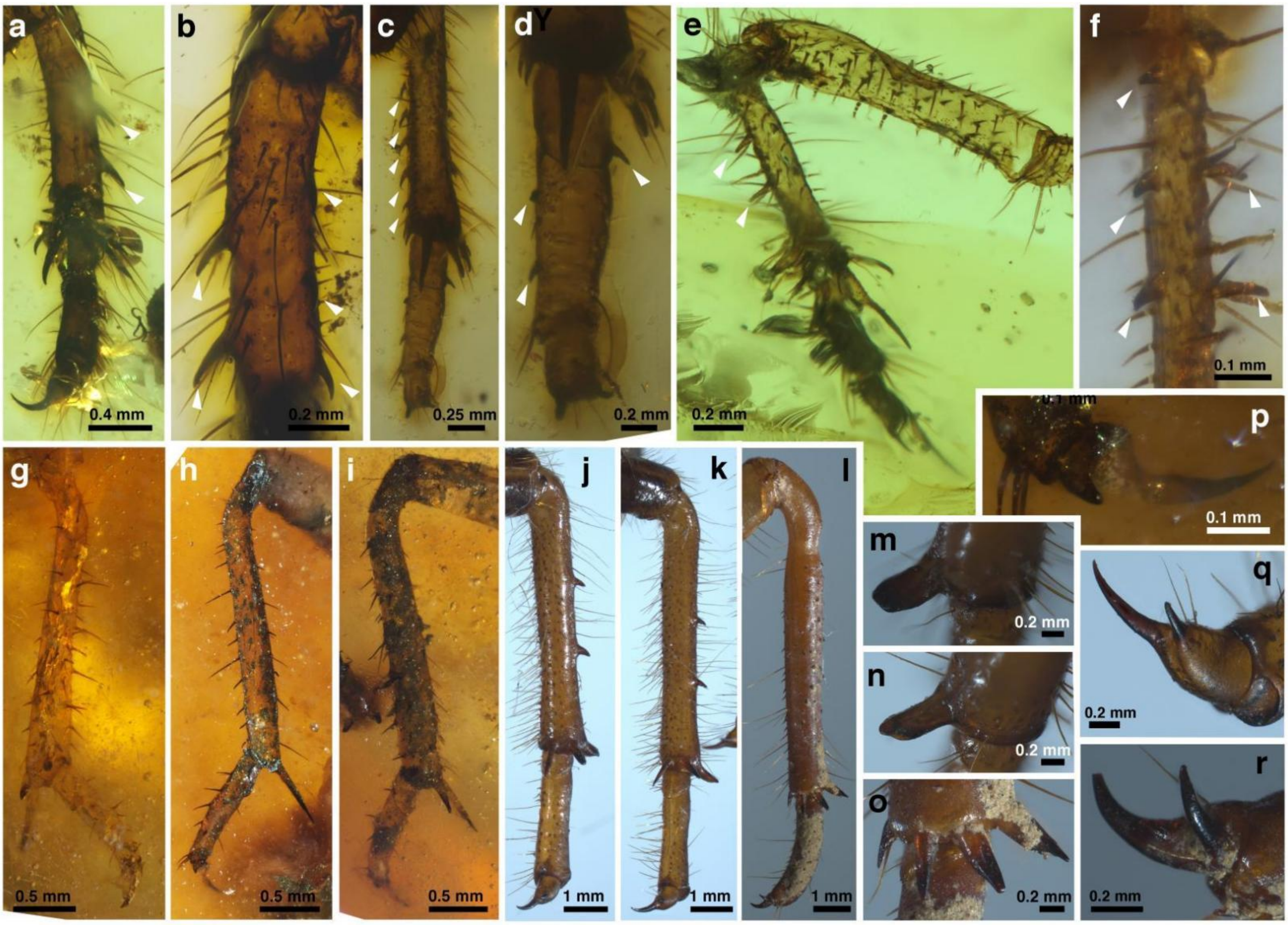

Supplementary Figure 15. Comparisons of midlegs and hindlegs of final-instar cicadoid exuviae from Kachin amber and extant species. a–**s**, Reflected light micrographs. **a**–**d**, Legs of sp. 2 from Kachin amber. **a**, Left midleg. **b**, Tibia in (a). **c**, Left hindleg**. d**, Apical spines on tibia, tarsus and pretarsi in (c)**. e**–**f**, Leg of sp. 3 (probably a midleg) in Kachin amber. **f**, Tibia in (e). **g**–**i**, Legs of sp. 4 in Kachin amber. **g**, Left midleg. **h**, Right midleg. **i**, Right hindleg. **j**–**k**, Mid- and hindlegs of extant *T. tomentosa*. **j**, Left midleg. **k**, Left hindleg. **l**, Left hindleg of extant *H. maculaticollis*. **m**–**n**, Flat apical spines of meso and meta tibia in (j) and (k), respectively. **o**, Apical spines of metatibia in (l). **p**, Pretarsi in (h). **q**, Pretarsi in (k). **r**, Pretarsi in (l).

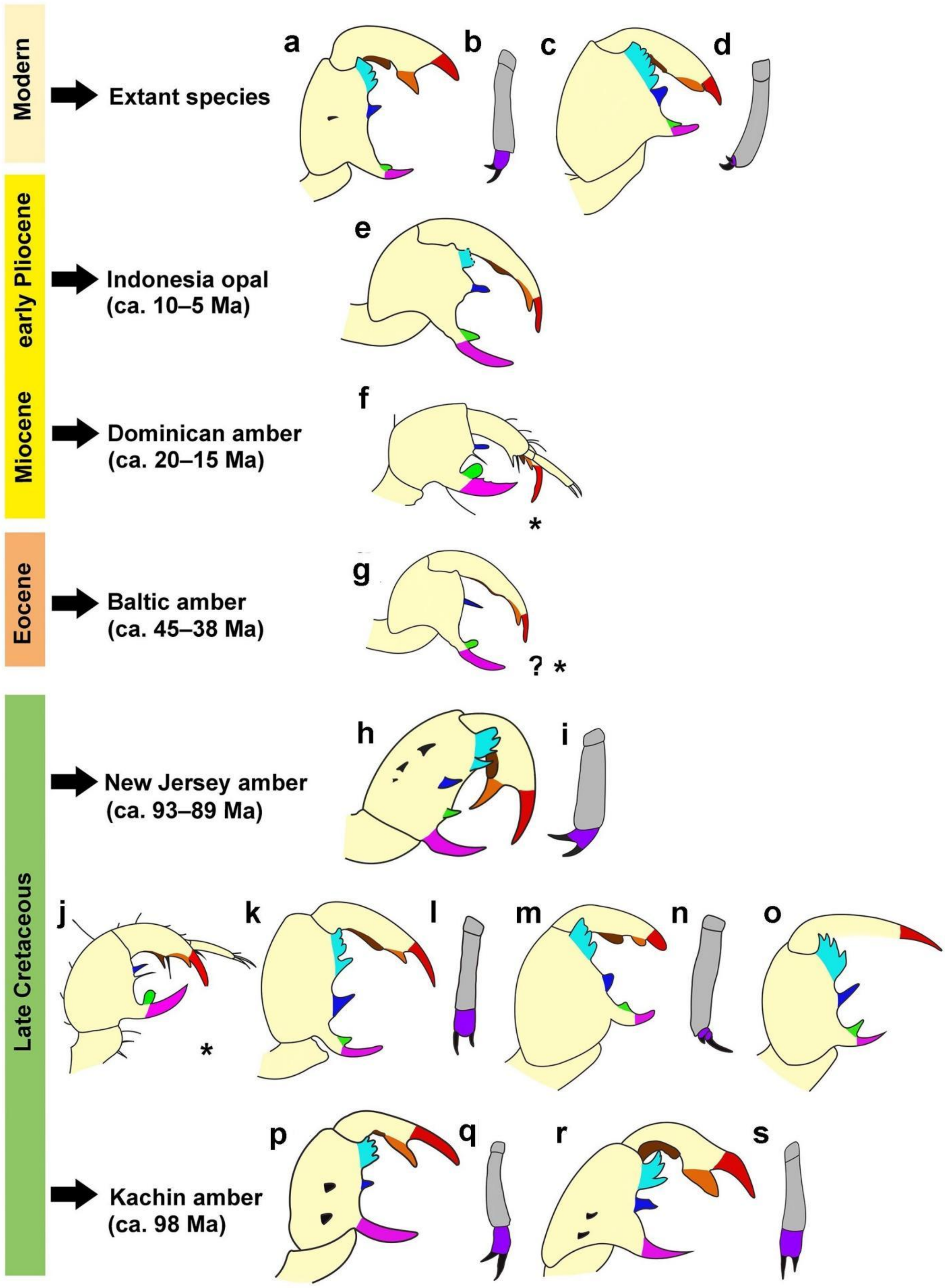

Modern
Extant species
early Pliocene
Indonesia opal
(ca. 10–5 Ma)
Miocene
Dominican amber
(ca. 20–15 Ma)
Eocene
Baltic amber
(ca. 45–38 Ma)
Late Cretaceous
New Jersey amber
(ca. 93–89 Ma)
Kachin amber
(ca. 98 Ma)
a
b
c
d
e
f
g
h
i
j
k
l
m
n
o
p
q
r
s
*
? *

Supplementary Figure 16. Schematic line drawings showing forelegs in nymphs of Cicadoidea from mid-Cretaceous to the present. **a**, **b**, Foreleg and pretarsus of *Tettigarcta tomentosa*. **c**, **d**, Foreleg and pretarsus of *Hyalessa maculaticollis*. **e**, Foreleg of Pliocene (uncertain stage) nymph from Indonesian opal. The illustration referenced based on the CT data from Chauviré et al., 2020. **f**, Foreleg of *Dominicicada youngi* in Dominican amber referenced and modified from Poinar Jr and Kritsky, 2012. **g**, Foreleg of a first-instar nymph in Baltic amber referenced from Shcherbakov, 2002. **h**, **i**, Foreleg and pretarsus of final-instar nymph in Late Cretaceous New Jersey amber referenced and modified from Grimaldi and Engel, 2005. **j**, Foreleg of *Burmacicada protera* in mid-Cretaceous amber referenced and modified from Poinar Jr and Kritsky, 2012. **k**, **l**, Nymphal sp. 1, final-instar nymph (NIGP201898). **m**, **n**, Nymphal sp. 4 (NIGP201900), exuviae. **o**, Nymphal sp. 5 (NIGP201901), exuviae. **p**, **q**, Nymphal sp. 2 (MGM2016–0.17), exuviae. **r**, **s**, Nymphal sp. 3 (LYU–BC2004), exuviae. Asterisks represent first-instar nymphs. The question mark represents uncertainty about the morphology. Asterisks represent first-instar nymphs. Each colour in the figure represents a different structure, and these colour-structure associations remain consistent throughout the figure.

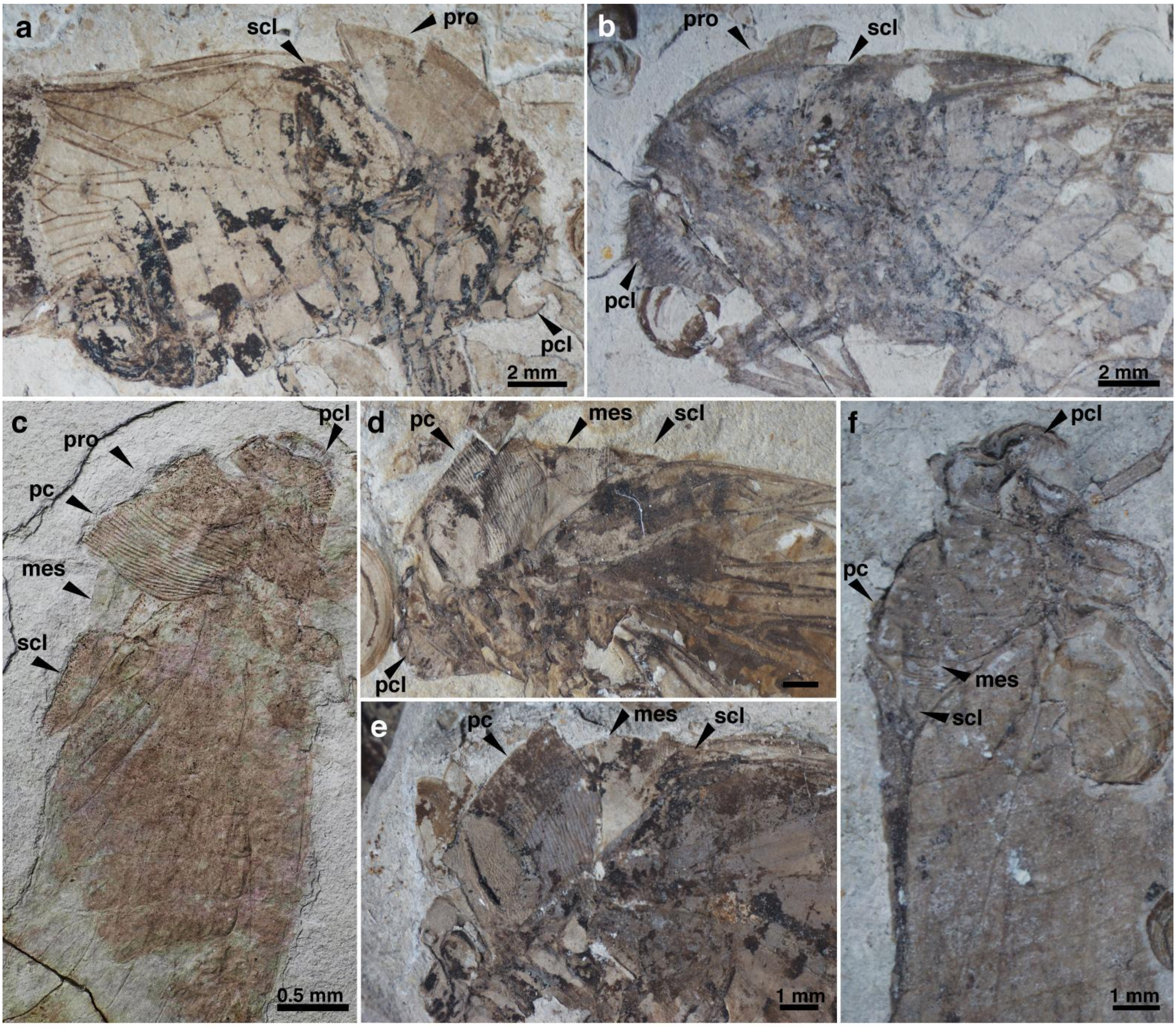

Supplementary Figure 17. Cicadoidea fossils from the middle Late Jurassic Yanliao Biota at Daohugou, Inner Mongolia, China. a–**b**, Fossils showing pronotal characters including without wrinkled collar, and pronotum concealing all parts of the mesonotum except scutellum. **a**, NIGP156691. **b**, NIGP156705a. **c**–**f**, Fossils showing pronotal characteristics including a wrinkled pronotal collar, in which the pronotum does not conceal all part of the mesonotum, except the scutellum. **c**, NIGP151861b. **d**, NIGP156712. **e**, NIGP156709. **f**, NIGP156708. Abbreviations: pc, pronotal collar; pcl, postclypeus; pro, pronotum; mes, part of mesonotum except scutellum; and scl, scutellum. All specimens are deposited at NIGPAS.

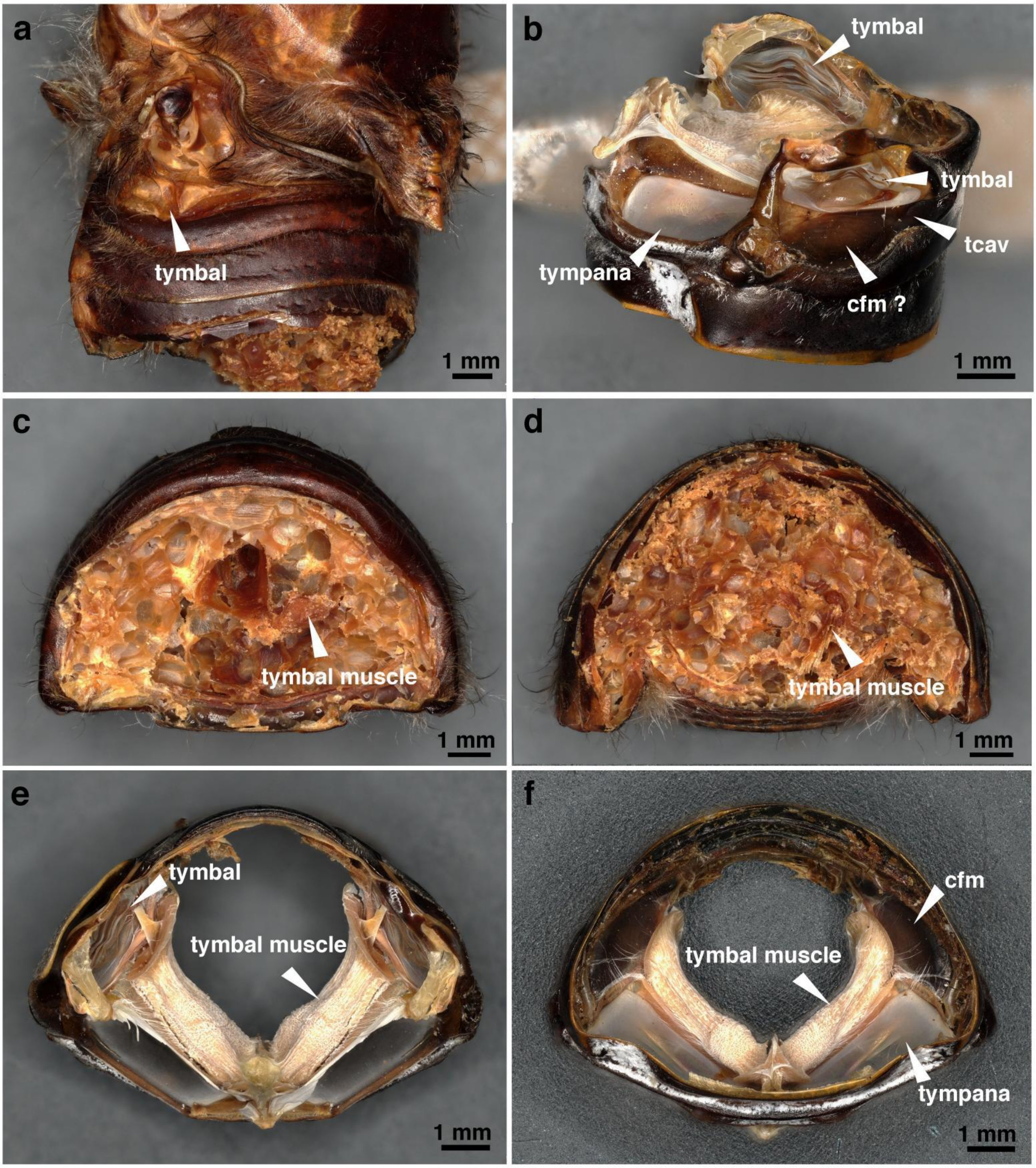
a
tymbal
1 mm
b
tymbal
tymbal
tcav
tympana
cfm ?
1 mm
c
tymbal muscle
1 mm
d
tymbal muscle
1 mm
e
tymbal
tymbal muscle
1 mm
f
cfm
tymbal muscle
tympana
1 mm

Supplementary Figure 18. Abdominal structures associated with the tymbal organs of living species. a, Lateral view *Tettigarcta crinite* (Tettigarctidae). **b**, Lateral view of *Platypleura kaempferi* (Cicadidae). **c**, **d**, Front and rear views of the transverse section of the second abdominal segment of *T. crinite*, respectively. **e**, **f**, Rear view of transverse section of the second segment of the abdomen of *Pl. kaempferi*, respectively. Abbreviation: cfm, chitinous thickened membrane; tcav, tmbal cavity.

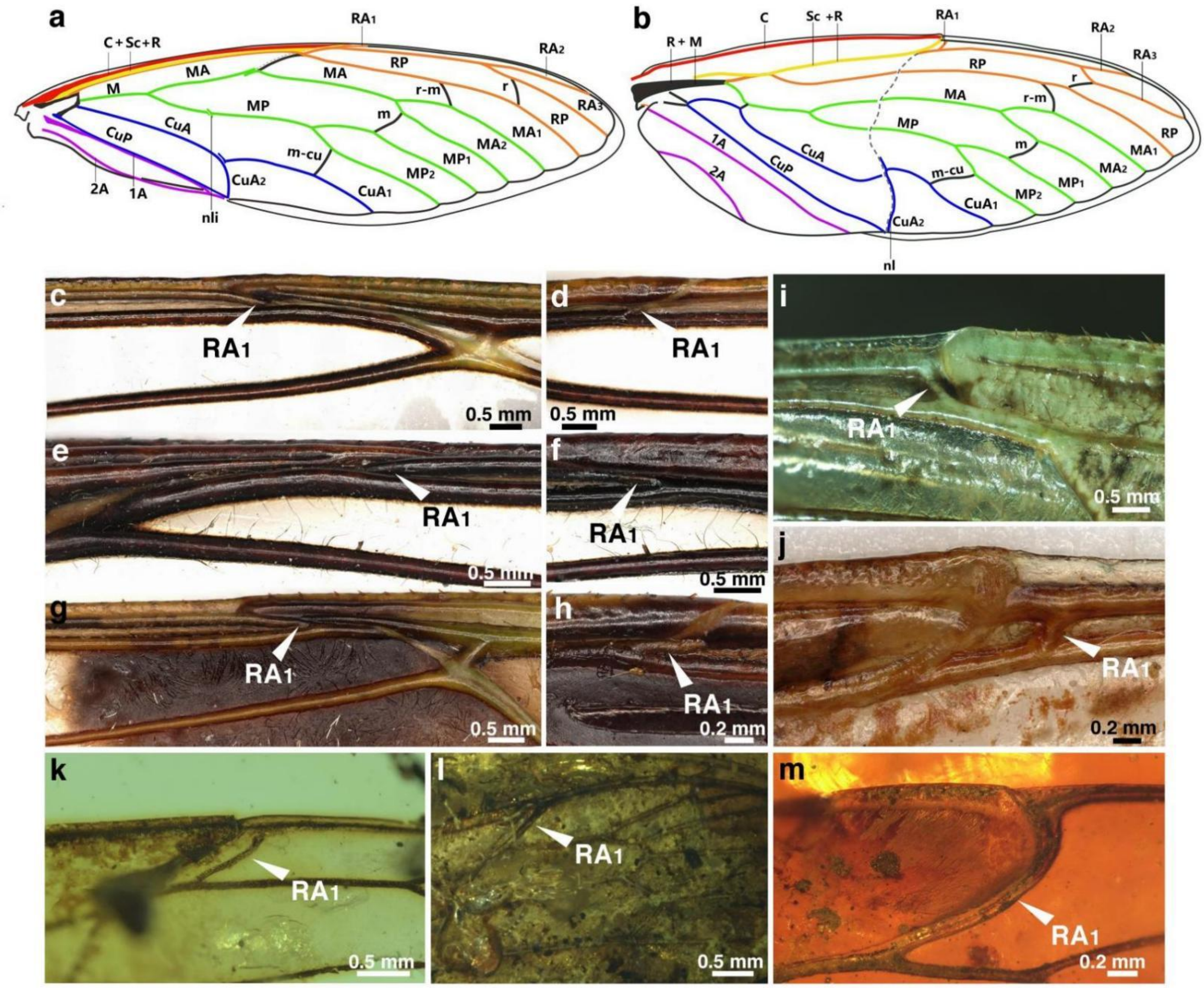

Supplementary Figure 19. Description scheme outlining the forewing venation of Cicadoidea in this study. a, Forewing venation of *Hyalessa maculaticollis* (Cicadidae). **b**, Forewing venation of *Tettigarcta crinita* (Tettigarctidae). Each colour in the image represents different veins, and these colour-structure associations remain consistent throughout the figure with accompanying labels. **c–j**, Magnification of vein RA1 from extant species and fossils. **c**, **d**, Showing the RA_1 vein from *Cryptotympana atrata*. **d**, Opposite surface of (c). **e**, **f**, Showing the RA_1 vein from *H. maculaticollis*. **f**, Opposite surface of (e). **g**, **h**, Showing the RA_1 vein from *Pl. kaempferi*. **h**, Opposite surface of (G). **i**, Showing the RA_1 vein from *T. tomentosa*. **j**, Showing the RA_1 vein from *T.*

crinita. **k**, Showing the RA_1 vein from *C. shcherbakovi*. **l**, Showing the RA_1 vein from *C. shcherbakovi*. **m**, Showing the RA_1 vein from *V. parallelica*.

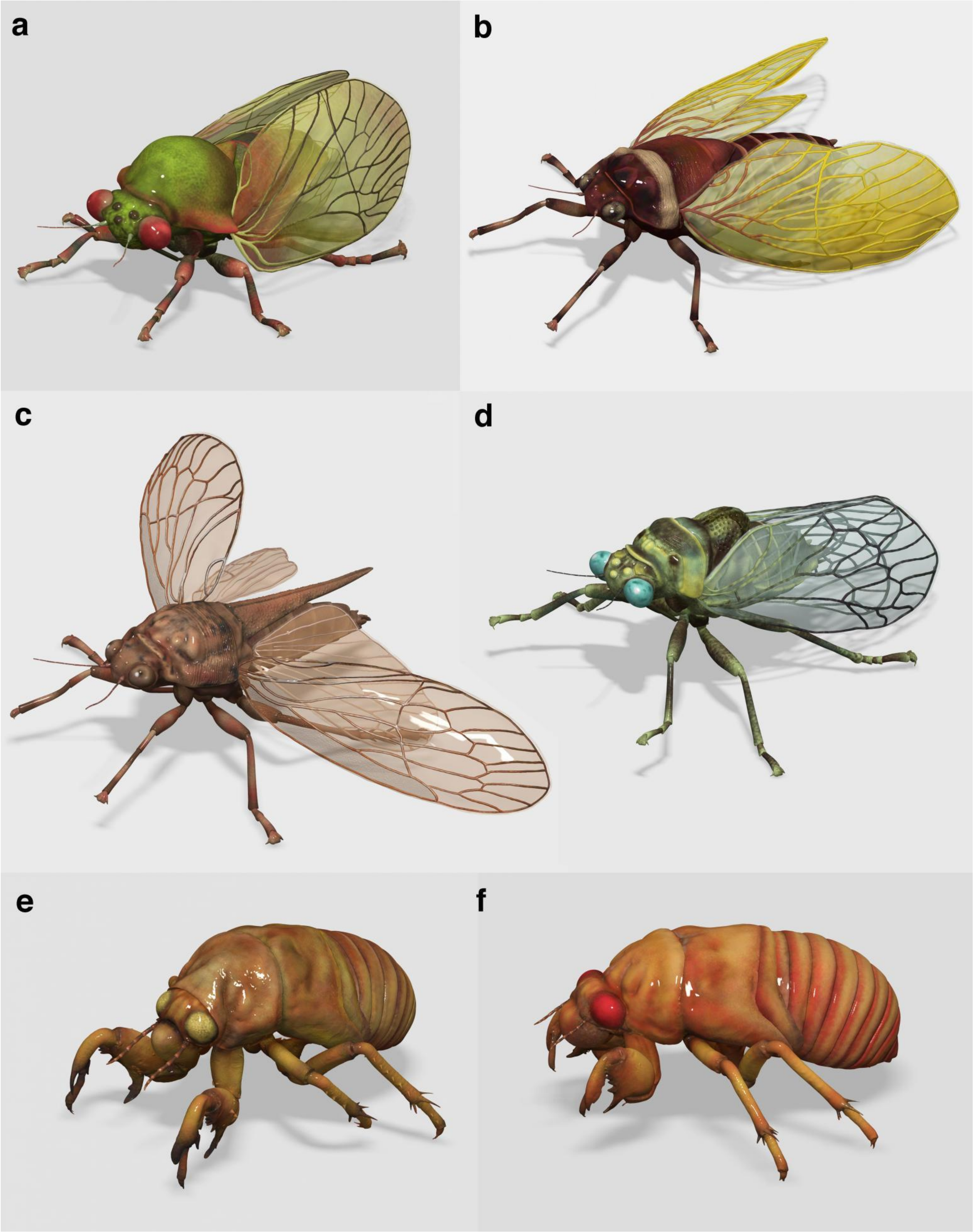
a
b
c
d
e
f

Supplementary Figure 20. Individual reconstructions of the fossils. The reconstructions are reconstruction by Mr. Dinghua Yang. **a**, Reconstruction of *Eunotalia* gen nov. **b**, Reconstruction of *Vetuprosbole*. **c**, Reconstruction of *Cretotettigarcta*. **d**, Reconstruction of *Pranwanna* gen nov. **e**, Reconstruction of the nymph represented by sp. 2 and sp. 3. **f**, Reconstruction of the nymph represented by sp.1, sp.2 and sp. 5.

Supplementary Table 1.

The description of each landmark for the geometric morphometrics analysis (GMA) of the dorsal profile of the head and thorax referring to Fig. 3b and Supplementary Fig. 9.

Number	Position
1	Right side of left eye
2	Left side of right eye
3	The middle of most anterior margin of pronotum
4	The middle of anterior fissure of pronotal collar
5	The middle of posterior margin of pronotum
6	The middle of posterior margin of mesonotum before scutellum (close to the middle of scutal depressions)
7	The distal ends of scutellum or left lowest point of cruciform elevation
8	The distal ends of scutellum or right lowest point of cruciform elevation
9	Right lateral angle of pronotum
10	Left lateral angle of pronotum

Supplementary Table 2.

Eigenvalues and contributions of the first five principal components of landmarks for thg principal component analysis (PCA) of the dorsal profile of the head and thorax referring to Fig. 3b.

Principal Component Number	Eigenvalue	Percentage of Variance (%)	Cumulative (%)
1	0.02826	63.2	63.2
2	0.00668	14.9	78.1
3	0.00294	6.6	84.7
4	0.00183	4.1	88.8
5	0.00153	3.4	92.2

Supplementary Table 3.

Eigenvector matrix of the principal components 1, 2 and 3 of the dorsal profile of the head and thorax with 10 landmarks of 175 cicadoid specimens referring to Fig. 3b.

Number	PC1	PC2	PC3
X1	0.51569	0.13351	-0.1694
Y1	0.38995	0.03549	0.36781
X2	0.52437	0.14281	-0.15249
Y2	-0.39017	-0.04867	-0.38536
X3	0.49792	0.10033	0.00574
Y3	0.01475	-0.00349	0.01743
X4	0.18379	0.08369	0.03187
Y4	0.01308	0.00525	0.00748
X5	-0.01116	0.06744	0.01636
Y5	0.03009	0.00247	0.00687
X6	0.11531	-0.20382	-0.13142
Y6	-0.11473	0.02599	0.01183
X7	-0.90715	0.01788	0.20661
Y7	0.22149	0.03088	0.1772
X8	-0.90502	0.02436	0.21087
Y8	-0.17829	-0.03754	-0.16658
X9	-0.00479	-0.19417	-0.02207
Y9	0.6582	-0.15292	0.12788
X10	-0.00897	-0.17204	0.00394
Y10	-0.64437	0.14253	-0.16456

Supplementary Table 4.

Descriptions of each landmark for the geometric morphometrics (GMA) analysis of forewing profiles referring to Supplementary Fig. 11.

Number	Position
1	The basal of upper forewing margin
2	The distal of forewing
3	The middle of the upper margin
4	The middle between No.1 and No. 3
5	The middle between No.2 and No. 3
6	The middle between No.1 and No. 4
7	The lowest of the Cubitus margin
8	The middle between No.2 and No. 8
9	The middle between No.7 and the basal of lower forewing margin

Supplementary Table 5.

Eigenvalues and contributions of the first seven principal components of landmarks of PCA referring to Supplementary Fig. 10a.

PC	Eigenvalue	% Variance	Cumulative (%)
1	0.00259	49.36	49.361
2	0.00097	18.45	67.814
3	0.00052	9.98	77.7928
4	0.00028	5.40	83.1884
5	0.00021	4.06	87.2508
6	0.00017	3.29	90.5414
7	0.00015	2.82	93.3581

Supplementary Table 6.

Eigenvector matrix of the principal components 1 and 2 of the forewing profiles with 9 landmarks of 238 cicadoid specimens referring to Supplementary Fig.10a.

Number	PC1	PC2	Number	PC1	PC2
X1	-0.24406	0.21144	Y5	0.0071589	0.26074
Y1	-0.18648	0.057431	X6	-0.04278	0.19284
X2	0.13333	0.017238	Y6	-0.16732	0.099384
Y2	-0.018765	0.23821	X7	-0.62531	-0.42061
X3	0.41107	-0.087276	Y7	0.1489	-0.48252
Y3	0.015734	0.28243	X8	-0.2406	-0.12711
X4	0.10139	0.13763	Y8	0.11041	-0.1853
Y4	-0.13038	0.15964	X9	0.23537	0.032434
X5	0.27159	0.043413	Y9	0.22074	-0.43003

Supplementary Table7.

Characters of the nymphal forelegs for principal coordinate analysis (PCoA) referring to Fig. 3d.

Number	Characteristics
1	Whether secondary apical tooth of tibia length exceeds half of the apt length: (0) not beyond: (1) beyond
2	Whether a short distance is present between pbt and apt: (0) no; (1) yes
3	The number of femoral combs: (0) three; (1) four or five; (2) general more than five
4	The front tooth of the femoral comb: (0) tooth independent; (1) aggregated into a flat plate
5	Whether there is an accessory tooth of femur: (0) yes; (1) no
6	Whether femur surface bears spines: (0) yes; (1) no
7	The number of the spines on the femur surface: (0) none; (1) two; (2) one
8	Pretarsi: (0) enlarged; (0) not enlarged
9	Metatarsi: (0) without lateral spines; (0) with lateral spines

Supplementary Table 8.

Eigenvalues and contributions of the first three principal coordinates referring to Fig. 3d.

PC	Eigenvalue	%Percent
1	0.74982	76.739
2	0.16888	17.284
3	0.0074379	0.76123

References

1. Breitkreuz, L. C., Winterton, S. L. & Engel, M. S. Wing tracheation in Chrysopidae and other Neuropterida (Insecta): a resolution of the confusion about vein fusion. *Am. Mus. Novit.* **2017**, 1–44 (2017).
2. Chauviré, B. et al. Arthropod entombment in weathering-formed opal: new horizons for recording life in rocks. *Sci. Rep.* **10**, 1–9 (2020).
3. Chen, J. et al. A new bizarre cicadomorph family in mid-Cretaceous Burmese amber (Hemiptera, Clypeata). *Cretac. Res.* **97**, 1–15 (2019).
4. Chen, J. et al. Geometric morphometric analysis for the systematic elucidation of new Hylicellidae from the Jurassic of China (Hemiptera: Cicadomorpha). *J. Paleontol.* **96**, 1119–1131 (2022).
5. Comstock, J. H. & Needham, J. G. The wings of insects. Chapter III. The specialization of wings by reduction. *Am. Nat.* **32**, 231–257 (1898).
6. Dworakowska, I. Main veins of the wings of Auchenorrhyncha (Insecta, Rhynchota: Hemelytrata). *Entomol. Abh.* **52**, 63–108 (1988).
7. Fu, Y. & Cai, C., Huang, D. First Mesozoic procercopids in mid-Cretaceous amber from northern Myanmar (Hemiptera: Cercopoidea). *Geol. Mag.* **157**, 506–512 (2020).
8. Gall, L. F. & Tiffney, B. H. A fossil noctuid moth egg from the Late Cretaceous of eastern North America. *Science* **219**, 507–509 (1983).
9. Grimaldi, D. & Engel, M. S. *Evolution of the Insects* (Cambridge University Press, 2005).
10. Hamilton, K. A. The insect wing, Part I. Origin and development of wings from not al lobes. *J. Kans. Entomol. Soc.* 421–433 (1971).
11. Jiang, H., Chen, J., Jarzembowski, E. & Wang, B. An enigmatic fossil hairy cicada (Hemiptera, Tettigarctidae) from mid-Cretaceous Burmese amber. *Cretac. Res.* **96**, 14–18 (2018).
12. Kukalová-Peck, J. in *The Insects of Australia*, CSIRO, Ed. (Cornell Univ. Press, Itha ca, NY, 1, 141–179, 1991).
13. Lambkin, K. J. *Mesodiphthera* Tillyard, 1919, from the Late Triassic of Queensland, the oldest cicada (Hemiptera: Cicadomorpha: Cicadoidea: Tettigarctidae). *Zootaxa* **4567**, 358–366 (2019).
14. Maccagnan, D. & Martinelli, N. M. Descrição das ninfas de *Quesada gigas* (Olivier)

(Hemiptera: Cicadidae) associadas ao cafeeiro. *Neotrop. Entomol.* **33**, 439–446 (2004).

15. Moulds, M. S. *Australian cicadas* (University of New South Wales Press,1990).
16. Moulds, M. S. An appraisal of the higher classification of cicadas (Hemiptera: Cicadoidea) with special reference to the Australian fauna. *Rec. Aust. Mus.* **57**, 375–446 (2005).
17. Moulds, M. S. Cicada fossils (Cicadoidea: Tettigarctidae and Cicadidae) with a review of the named fossilised Cicadidae. *Zootaxa* **4438**, 443–470 (2018).33
18. Moulds, M. S., Frese, M. & McCurry, M. R. New cicada fossils from Australia (Hemiptera: Cicadoidea: Cicadidae) with remarkably detailed wing surface nanostructure. *Alcheringa* **46**, 1–13 (2022).
19. Nel, A. et al. Traits and evolution of wing venation pattern in paraneopteran insects. *J. Morphol.* **273**, 480–506 (2012).
20. Poinar, Jr, G. & Kritsky, G. Morphological conservatism in the foreleg structure of cicada hatchlings, *Burmacicada protera* n. gen., n. sp. in Burmese amber, *Dominicicada youngi* n. gen., n. sp. in Dominican amber and the extant *Magicicada septendecim* (L.) (Hemiptera: Cicadidae). *Hist Biol.* **24**, 461–466 (2012).
21. Shcherbakov, D. The 270 million year history of Auchenorrhyncha (Homoptera). *Denisia* **176**, 29–36 (2002).
22. Shimmi, O., Matsuda, S. & Hatakeyama, M. Insights into the molecular mechanisms underlying diversified wing venation among insects. *Proc. R. Soc. B: Biol. Sci.* **281**, 20140264 (2014).
23. Simon, C. et al. Off-target capture data, endosymbiont genes and morphology reveal a relict lineage that is sister to all other singing cicadas. *Biol. J. Linn. Soc*. **128** (4), 865–886 (2019).
24. Qiao, D., Zhang, W., Zhang, X., Ren, D. & Yao, Y. New genus and species of Tettigarctidae (Hemiptera: Cicadomorpha: Cicadoidea) from the mid-Cretaceous amber of northern Myanmar. *Cretac Res*. **126**, 104900 (2021).
25. Hou, Z., Li, Q. & Wei, C. Morphology and identification of the final instar nymphs of three cicadas (Hemiptera, Cicadidae) in Guanzhong Plain, China based on comparative morphometrics. *Zookeys* **425**, 33–50 (2014).
26. Song, J. H., Kim, W. J., Cha, J. M., Yang, S., Choi, G. & Moon, B. C. Comparative morphological, ultrastructural, and molecular studies of four Cicadinae species using exuvial legs. *Insects* **10**, 199 (2019).
