# Supplementary material for: Mesozoic evolution of cicadas and their origins of vocalization and root feeding
Source: Nat Commun. 2024 Jan 8;15:376. doi: 10.1038/s41467-023-44446-x (PMC10774268; doi:10.1038/s41467-023-44446-x)
Supplement: Supplementary file 3 — Description of Additional Supplementary Files [file 41467_2023_44446_MOESM3_ESM.docx]

**Description of Additional Supplementary Files**

File Name: Supplementary Data 1

Description: Phylogenetic matrix referring to Fig. 3a and Supplementary Figure 8.

File Name: Supplementary Data 2

Description: NMDS matrix of forewing characters referring to Fig. 3c.

File Name: Supplementary Data 3

Description: Comparison of morphological characters among final-instar nymphs and exuviae of fossil and extant taxa.

File Name: Supplementary Data 4

Description: Fossil records of arthropod root-feeding.
